# Advancements and Challenges in Organic–Inorganic Composite Solid Electrolytes for All-Solid-State Lithium Batteries

**DOI:** 10.1007/s40820-024-01498-y

**Published:** 2024-09-20

**Authors:** Xueyan Zhang, Shichao Cheng, Chuankai Fu, Geping Yin, Liguang Wang, Yongmin Wu, Hua Huo

**Affiliations:** 1 State Key Laboratory of Space Power-Sources, School of Chemistry and Chemical Engineering, Harbin Institute of Technology, Harbin, 150001 People’s Republic of China; 2 College of Chemical and Biological Engineering, Zhejiang University, Hangzhou, 310058 People’s Republic of China; 3 State Key Laboratory of Space Power-Sources, 2965 Dongchuan Road, Minhang District, Shanghai, 200240 People’s Republic of China

**Keywords:** Composite solid electrolytes, Inorganic filler, Interfacial stability, Li-ion conduction mechanism, Characterization techniques

## Abstract

To address the limitations of contemporary lithium-ion batteries, particularly their low energy density and safety concerns, all-solid-state lithium batteries equipped with solid-state electrolytes have been identified as an up-and-coming alternative. Among the various SEs, organic–inorganic composite solid electrolytes (OICSEs) that combine the advantages of both polymer and inorganic materials demonstrate promising potential for large-scale applications. However, OICSEs still face many challenges in practical applications, such as low ionic conductivity and poor interfacial stability, which severely limit their applications. This review provides a comprehensive overview of recent research advancements in OICSEs. Specifically, the influence of inorganic fillers on the main functional parameters of OICSEs, including ionic conductivity, Li^+^ transfer number, mechanical strength, electrochemical stability, electronic conductivity, and thermal stability are systematically discussed. The lithium-ion conduction mechanism of OICSE is thoroughly analyzed and concluded from the microscopic perspective. Besides, the classic inorganic filler types, including both inert and active fillers, are categorized with special emphasis on the relationship between inorganic filler structure design and the electrochemical performance of OICSEs. Finally, the advanced characterization techniques relevant to OICSEs are summarized, and the challenges and perspectives on the future development of OICSEs are also highlighted for constructing superior ASSLBs.

## Highlights

- The lithium-ion conduction mechanism of organic-inorganic composite solid electrolytes (OICSEs) is thoroughly conducted and concluded from the microscopic perspective based on filler content, type, and system.
- The classic inorganic filler types, including inert and active fillers, are categorized with special emphasis on the relationship between inorganic filler structure design and the electrochemical performance of OICSEs.
- Advanced characterization techniques for OICSEs are discussed, and the challenges and prospects for developing superior all-solid-state lithium batteries are highlighted.

## Graphical abstract

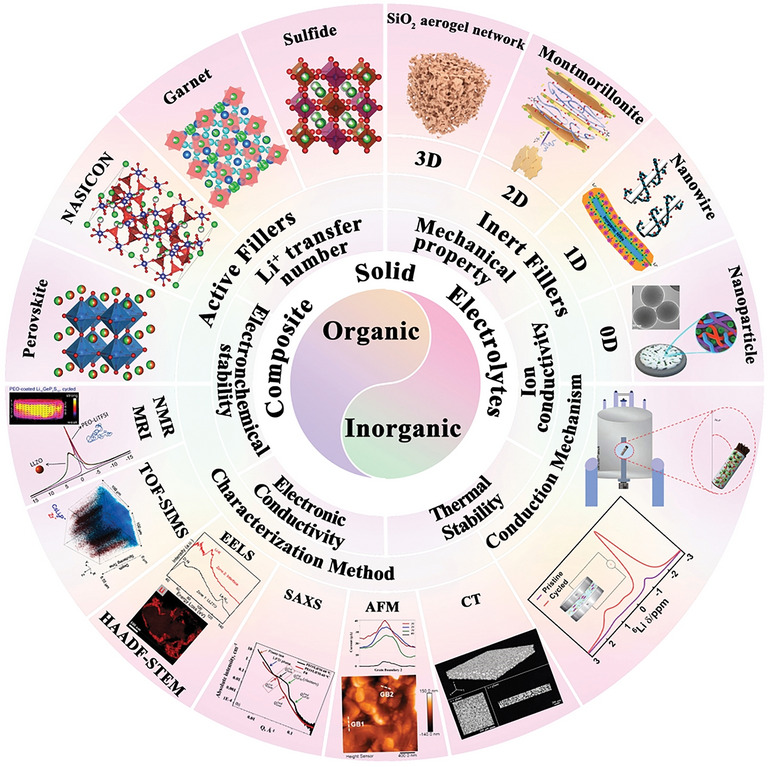

## Introduction

Rechargeable lithium-ion batteries (LIBs) are associated with significant safety concerns due to flammable and volatile organic liquid electrolytes, especially in large-scale energy storage applications such as electric vehicles and electronic devices [1–5]. In addition, the energy density of commercial lithium-ion batteries with liquid electrolyte and carbon-based anodes has reached 260 Wh kg^−1^, which is close to their theoretical limitation [6–8]. All-solid-state lithium metal batteries (ASSLBs), with super-high theoretical energy density (> 300 Wh kg^−1^) and excellent safety, have been widely recognized as one of the most promising next-generation battery technologies [9–11]. Solid-state electrolytes (SEs), as an important component of ASSLBs, have presented a rapidly increasing trend of investigations on SEs research in recent years [12–15].

The physicochemical properties of the SEs, including interfacial reaction kinetics, safety, and durability, are critical to ASSLBs [16–20]. SEs can be divided into inorganic solid electrolytes (ISEs) and organic solid electrolytes (OSEs). ISEs exhibit high ionic conductivity (10^−4^–10^−3^ S cm^−1^), Li^+^ transference number (~ 1), excellent thermal stability, and ultra-high mechanical strength [21, 22]. However, the inherent fragility and high hardness often result in poor interfacial wettability with both the cathode and anode and significantly increased processing challenges. Therefore, the practical application of ISEs still faces uncertainty [23–25]. By contrast, OSEs show higher feasibility with excellent elasticity, well flexibility, superior interface adhesion, and relatively high compatibility [26–29]. However, the polymer matrix with high crystallinity at room temperature (RT) always results in low ionic conductivity (10^−7^–10^−5^ S cm^−1^), which is unfavorable for achieving high power density. Furthermore, the thermodynamic instability (oxidation potential less than 4 V vs. Li^+^/Li) restricts the matching with high-voltage cathode materials, while relatively inferior mechanical properties struggle to inhibit the lithium dendrite formation and growth [30–32]. In this situation, numerous strategies have been employed to enhance the overall performance of OSEs, such as block/cross-linked copolymerization, incorporation of plasticizers, and addition of inorganic fillers [33–35]. Among these approaches, the organic–inorganic composite solid electrolytes (OICSEs), which integrate the advantages of the organic polymer and inorganic fillers, are widely considered the most simple and feasible method to develop high-performance SEs for ASSLBs [36–38].

Generally, the inorganic materials can be divided into two categories: inert materials [39–43] (e.g., metal oxides (Al_2_O_3_, SiO_2_, BaTiO_3_, TiO_2_, and MgO), halloysite nanotubes (HNTs), carbon materials (such as GO)), and active materials [44–46] (e.g., sulfide-type (Li_10_GeP_2_S_12_ (LGPS)), garnet-type (Li_7_La_3_Zr_2_O_12_ (LLZO), and NASICON-type (Li_1.3_Al_0.3_Ti_1.7_(PO_4_)_3_ (LATP)), and perovskite-type (Li_0.33_La_0.557_TiO_3_). It has been well confirmed that the functional mechanism of inorganic fillers can be summarized in the following three aspects [47–49]: (1) Inorganic fillers can improve the ratio of amorphous regions and enhance the mobility of local chain segments by disrupting the polymer crystallization behavior and reducing the glass transfer temperature (T_g_). (2) The special functional groups on the surface of fillers can couple with lithium salt anions or polymer matrix via Lewis acid–base interactions, thereby facilitating the lithium ion transfer behaviors. Several factors, including size, type, concentration, morphology, and surface modifications of fillers, influence the strength of these interactions. (3) The inorganic fillers can increase the Li^+^ transfer number of OICSE and inhibit the enrichment of anions on the anode side, thus enhancing the electrochemical stability of OICSE. (4) Well-dispersed inorganic fillers can also improve the mechanical strength and thermal stability of OICSEs, effectively improving the reliability and security of the battery system. To improve the electrochemical performance of OICSEs, various inorganic fillers with different dimensions, such as 0D particles, 1D nanowires, 2D nanosheets, and 3D networks, have been specifically designed and widely investigated [50–52]. These fillers, exhibiting diverse morphologies, can provide long-range transport channels for lithium ions, resulting in a rapid ion transport pathway between the cathode and anode [53, 54]. Active fillers can directly participate in ion transport compared to inert fillers due to their intrinsic ionic conductivity. Meanwhile, a percolation pathway with fast ionic conductivity between active filler and polymer matrix can be constructed in the OICSEs, which is beneficial to improve the electrochemical performance of the battery system [55, 56].

**Fig. 1:**
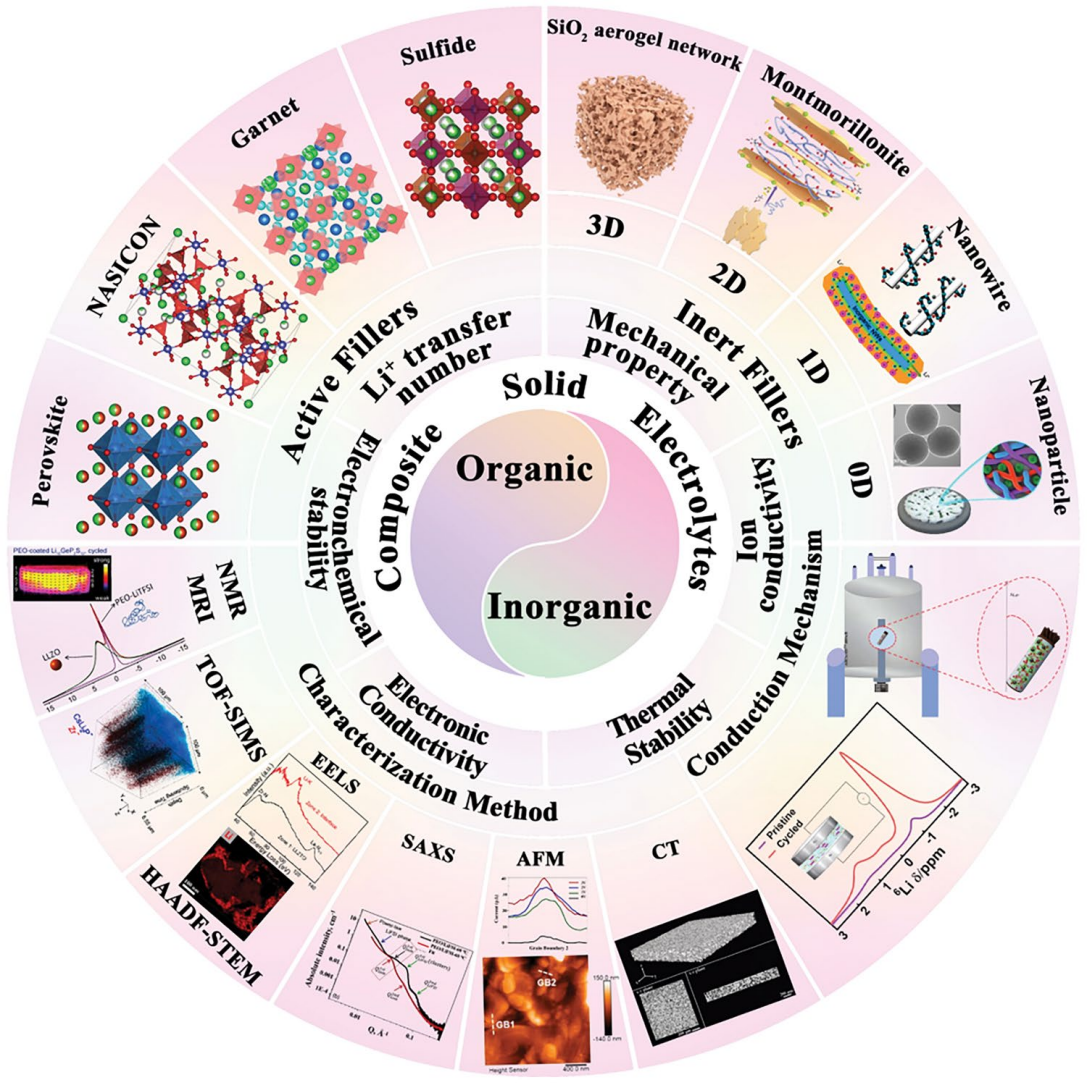
Scope and content diagram are discussed in this review

Here, we emphasize the significance of various inorganic filler types and advanced structures in optimizing the performance of OICSEs (Fig. 1). Initially, key parameters such as ionic conductivity, Li^+^ transference number, mechanical properties, electrochemical stability, electronic conductivity, and thermal stability are extensively investigated. Subsequently, the impacts of the size, content, shape, and arrangement of inorganic fillers on ionic conductivity are analyzed. In addition, the lithium-ion conduction mechanism of OICSE is thoroughly conducted and concluded from the microscopic perspective based on filler content, type, and system. Furthermore, the classic inorganic filler types, including both inert and active fillers, are categorized. Special emphasis is placed on the relationship between inorganic filler structure design and the electrochemical performance of OICSEs. Finally, Advanced characterization techniques for OICSEs like solid-state nuclear magnetic spectroscopy (NMR), magnetic resonance imaging (MRI), time-of-flight secondary ion mass spectrometry (TOF–SIMS), high-angle annular dark field scanning transmission electron microscopy (HAADF-STEM), electron energy loss spectroscopy (EELS), small-angle X-ray scattering (SAXS), X-ray computed tomography (CT), and atomic force microscopy (AFM) are discussed, along with their applications and future challenges. The review concludes with a summary and perspective, offering valuable insights to facilitate the research and development of OICSEs with appreciable overall performance.

## Key Parameters to Evaluate the Performance of OICSEs

The ionic conductivity, Li^+^ transference number, mechanical properties, electrochemical stability, electronic conductivity, and thermal stability are essential indicators for evaluating the performance of OICSEs. A common problem of OSEs is low ionic conductivity and insufficient mechanical strength, which restricts their practical application in ASSLBs. To overcome these problems, researchers incorporate inorganic fillers into the polymer matrix. These fillers not only enhance the ionic conductivity but also improve the mechanical strength of the electrolytes, thereby optimizing the overall electrochemical performance of OICSEs in ASSLBs.

### Ionic Conductivity

#### Effects of Inorganic Fillers on Ionic Conductivity

**Fig. 2:**
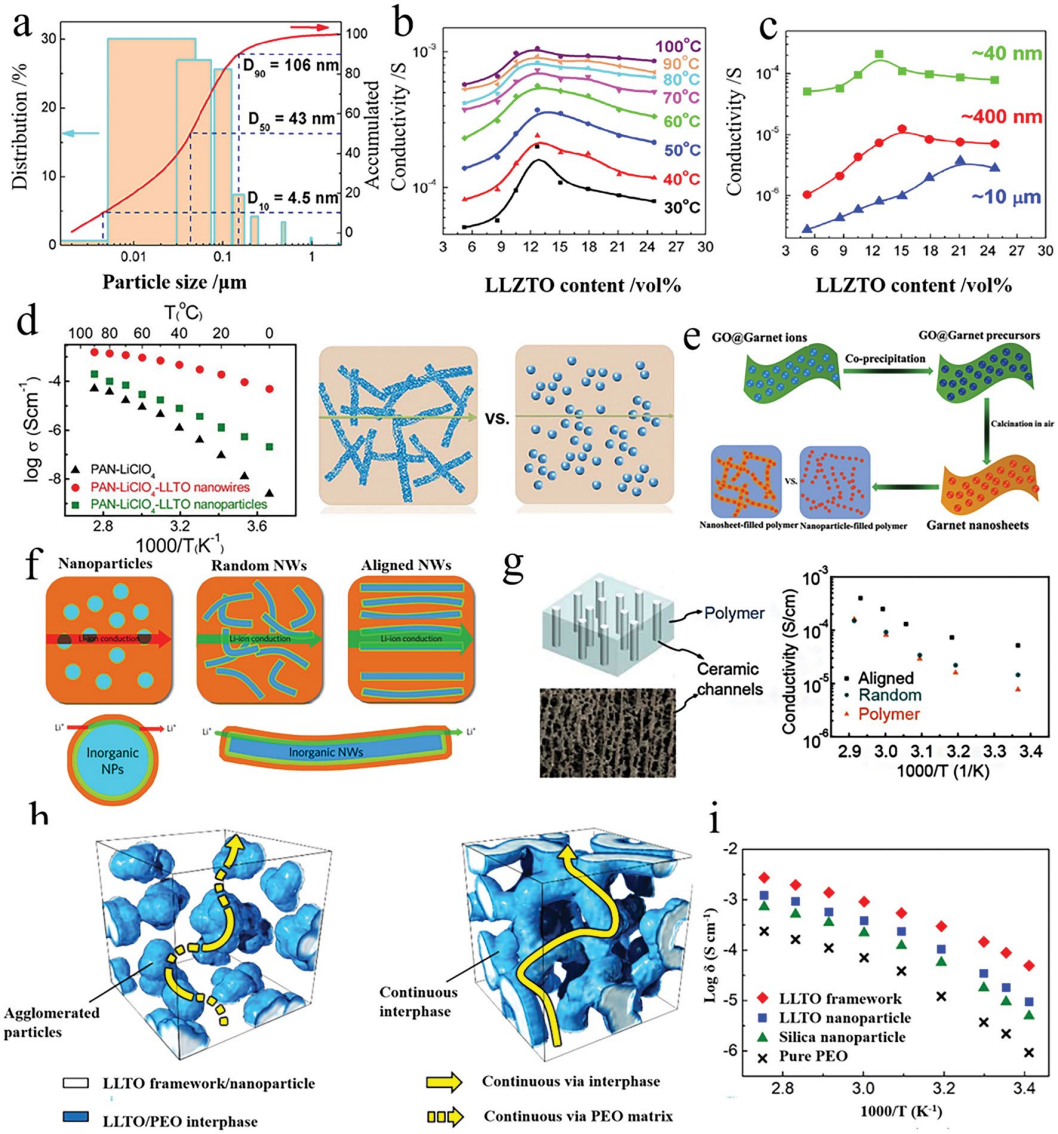
**a** Size distribution of LLZTO nanoparticles determined by a laser particle size analyzer. **b** Ion conductivities of PEO: LLZTO membranes with different volume fractions of LLZTO in size of D_50_ = 43 nm. **c** Ionic conductivity as a function of LLZTO volume fraction for LLZTO particles with different sizes [61], Copyright 2016, Elsevier. **d** Ion conductivities of PAN/LiClO_4_, PAN/LiClO_4_ with LLTO nanowires, and LLTO nanoparticles and the comparison of possible lithium-ion conduction pathway in nanowire-filled and nanoparticle-filled composite electrolytes [64], Copyright 2015, American Chemical Society. **e** Schematic diagram of garnet nanosheets and comparing composite electrolytes consisting of garnet nanoparticles [66], Copyright 2019, American Chemical Society. **f** Li-ion conduction pathways in OICSEs with nanoparticles, random nanowires, and aligned nanowires [67], Copyright 2017, Springer Nature Limited. **g** Ionic conductivity of vertically aligned, random, and polymer [68], Copyright 2017, American Chemical Society. **h** Schematics of agglomerated nanoparticles and 3D continuous framework. **i** Ionic conductivity of LLTO framework, LLTO nanoparticle, and silica particle OICSEs [69], Copyright 2018 Wiley

*(a) Particle Size and Content*: The inorganic particle size and content are key factors to improve the ionic conductivity of the OICSEs [57–59]. Dissanayake et al. evaluated the thermal and electrical properties of the (PEO)_9_LiCF_3_SO_3_-Al_2_O_3_ incorporating alumina filler grains with different specific surface areas [60]. The results indicate that the nano-porous alumina grains with 5.8 nm pore size and 150 m^2^ g^−1^ specific area and 15 wt% filler exhibited the maximum ionic conductivity, which is attributed to Lewis acid–base interactions of ionic species with O/OH groups on the filler surface. Generally, incorporating 10–20 wt% of ceramic filler into the polymer matrix is considered the optimal concentration for OICSEs. The particles tend to undergo agglomeration behavior with the increase of content, reducing the volume fraction and disrupting the percolation network at the interface. Zhang et al. investigated that Li-salt-free PEO and LLZTO nanoparticles in size of D_50_ = 43 nm show the highest ionic conductivity of 2.1 × 10^−4^ S cm^−1^ at 30 °C [61], which is nearly two orders of magnitude higher than that of micron-sized LLZTO fillers (Fig. 2a). When the LLZTO size is fixed at 43 nm, and content is 12.7 vol% within different temperature ranges (Fig. 2b), the PEO:12.7 vol% LLZTO membrane achieved the maximum ionic conductivity. Moreover, the result found that as the size of LLZTO particles increased from 40 to 400 nm and 10 mm, the optimal ceramic content also increased from 12.7 to 15.1 vol% and 21.1 vol%, respectively (Fig. 2c). Therefore, it can be concluded that the particle size is related to the percolation of LLZTO particles, and the percolation threshold decreases with the decrease in particle size [62]. Nanoparticles have a larger specific surface area and can increase the area of the polymer electrolyte/filler interface, providing more ion transport pathways, thus significantly increasing the ionic conductivity of the OICSEs [63]. Therefore, nanoparticles are more effective in improving ionic conductivity than micron-sized particles.

*(b) Shape of Inorganic Fillers*: The ion conduction mechanism based on the percolation effect has shown that the development of specially shaped ceramic fillers (nanowire fillers) can effectively improve the uniform transport of lithium ions and avoid the decrease of ionic conductivity caused by the agglomeration of fillers. Liu et al. explored the impact of nanoparticles and nanowires on the electrochemical performance of PAN/LiClO_4_ polymer electrolytes [64]. The 15 wt% LLTO nanowires would increase the ionic conductivity by three orders of magnitude over the same content of LLTO nanoparticles. This is mainly because LLTO nanowires create a longer distance than nanoparticles for the ion transport pathway (Fig. 2d). To improve the transport pathway of LLTO nanoparticles in the polymer matrix, Fu et al. reported a 3D garnet nanofiber network by electrostatic spinning and high-temperature annealing with an ionic conductivity of 2.5 × 10^−4^ S cm^−1^ at RT, two orders of magnitude higher than that of PEO-based electrolyte containing LLZO nanoparticles [65]. The increased conductivity can be attributed to the 3D interconnected structure, which offers a continuous transport pathway for Li ions. Compared to nanoparticles, garnet nanosheets also have interconnected Li ion transport pathways. Song et al. introduced 15 wt% garnet nanosheets into the polymer matrix for the first time, the ionic conductivity achieved was 3.6 × 10^−4^ S cm^−1^ at RT (Fig. 2e) [66]. Therefore, introducing nanowires and nanosheets into the polymer matrix or constructing a three-dimensional or two-dimensional interconnection network structure can provide a continuous Li-ion transport pathway, thereby obtaining higher ionic conductivity.

*(c) Arrangement of Inorganic Fillers*: To reduce the tortuosity of ion conduction pathway in OICSEs and to obtain larger inorganic particle/polymer interfaces, researchers have employed various methods to create OICSEs with oriented ceramic fillers, including electrostatic spinning, ice-templating-based methods, and 3D printing techniques. Liu and colleagues have developed well-oriented LLTO nanowires by electrospinning and embedding the LLTO nanowires in the PAN-LiClO_4_ electrolyte (Fig. 2f) [67]. This innovative design has resulted in a remarkable ionic conductivity of 6.05 × 10^−5^ S cm^−1^ at 30 °C, ten orders of magnitude higher than previous polymer electrolytes containing randomly arranged nanowires. The exceptional conductivity improvement is attributed to the efficient ion-conducting pathway created by the aligned nanowires. A flexible OICSE composed of vertically aligned and connected LATP NPs has been synthesized through the ice-templating process (Fig. 2g) [68]. The alignment of the nanoparticles creates direct channels for lithium ions, and the OICSEs show an impressive ionic conductivity of 0.52 × 10^−4^ S cm^−1^ at RT, which is 3.6 orders of magnitude higher than the PEO electrolyte containing LATP NPs randomly dispersed within the material. It has been discovered that 3D ceramic frameworks can significantly enhance the continuous and integrated ion-conduction network and increase mechanical strength. Bae and colleagues proposed a 3D hydrogel-derived nanostructured LLTO framework as a highly loaded nanofiller (Fig. 2h) [69]. The interconnected structure of the 3D LLTO framework provides a long-range, continuous pathway for Li-ions, which results in an impressive ionic conductivity of 8.8 × 10^−5^ S cm^−1^ at RT (Fig. 2i). Although OICSEs exhibit enhanced ionic conductivity, the polymer matrix limits the overall ionic conductivity. There are still many challenges to achieving practical applications at RT. Therefore, we need to improve the ionic conductivity further by designing the ceramic structure and optimizing the polymer matrix composition.

### Mechanical Properties

Mechanical properties are the physical characteristics of a material that it exhibits under the action of various forces, including toughness, hardness, strength, brittleness, and elasticity. Good mechanical properties can effectively hinder the formation and growth of lithium dendrites, which contribute to long cycling life [70]. Inorganic fillers in composite electrolytes usually have adequate strength but lack flexibility. The addition of inorganic fillers increases tensile strength but decreases elongation at break compared to the polymer matrix. For example, the addition of 5 wt% carbon nanotubes to the PEO polymer matrix can increase tensile strength by 160%, improving the mechanical properties of OICSEs significantly [71]. However, due to the presence of inorganic fillers, the polymer flexibility and adhesion in OICSEs decreases, which affects close contact with the electrodes and leads to an increase in interfacial resistance during cycling. Therefore, the balance between mechanical properties and interface contact with the electrode is required when designing OICSEs. The thickness of OICSEs is indeed crucial for the development of high-energy solid-state batteries. Currently, the thickness of the prepared OICSEs membranes is much thicker than that of commercial membranes, and most of the OICSEs are about 100 μm or thicker. It remains challenging to prepare OICSEs using traditional methods that maintain excellent mechanical properties while being ultrathin. Luo et al. [72] prepared ultrathin (4.2 μm) CSEs with a bilayer polymer structure (UFF/PEO/PAN/LiTFSI) by electrospinning, and the hard UFF ceramic scaffolds can maintain the mechanical strength. The elastic moduli of the PEO and PAN sides were measured by nanoindentation to be 298 and 1072 MPa, respectively. The high energy density of 506 Wh kg^−1^ and 1514 Wh L^−1^ is achieved based on LiNi_0.8_Co_0.1_Mn_0.1_O_2_ (NCM811) cathodes with a low N/P ratio and long lifespan over 3000 h. Wang et al. [73] fabricated LLZO layer and metal–organic framework (MOF) layer on both sides of polyethylene (PE) by tape casting and developed an ultrathin (12.6 μm) asymmetric composite solid electrolyte. The Li-symmetric battery has an ultra-long cycle (5000 h) and the assembled pouch cells provided a gravimetric/ volume energy density of 344.0 Wh kg^−1^/773.1 Wh L^−1^. However, it should be noted that OICEs inevitably reduce mechanical strength and increase the risk of membrane rupture or lithium dendrite growth, leading to interruption of ionic conduction and cell failure. Meanwhile, excessive hardness or elastic modulus may increase the impedance at the electrode–electrolyte interface, affecting the energy density and power density of the battery. Therefore, when designing and optimizing OICSEs, the above mechanical properties need to be considered to achieve excellent electrochemical performance and long-life battery systems.

Generally, the mechanical strength is described by the equations of Young's modulus of elasticity (E, MPa) and shear modulus (G, MPa). The specific equations are as follows [74, 75]:

1 $$E = {\text{V}\upiota }^{2} {{\rho }}\frac{{\left( {1 + {{\upnu }}} \right)\left( {1 - 2{{\upupsilon }}} \right)}}{{\left( {1 - {{\upnu }}} \right)}}$$

2 $$G = \frac{{\text{E}}}{{2\left( {1 + \upnu } \right) }}$$

where $\uprho$ is the density, ${\text{V}\upiota }$ is the longitudinal velocity, and $\upnu$ is Poisson’s ratio, ν = 0.257 [76]. In addition to Young's modulus and shear modulus, other parameters such as maximum stress (MPa) and strain at break (mm/mm) are also helpful in describing the mechanical properties of OICSEs in ASSLBs.

### Li-Ion Transference Number

Li-ion transference number is another important parameter to evaluate the electrochemical performance of OICSEs, which is the contribution of Li-ion transport charge to the total charge, calculated as the ratio of Li-ionic conductivity to total ionic conductivity. In OICSEs, which consist of multiple ions and are referred to as multi-ion conductors, the ionic conductivity is influenced by both Li-ion and anion transport. Lithium ions and anions can move during cycling but move in opposite directions. Consequently, a significant Li-ion concentration gradient is formed from the anode to the cathode, impeding Li-ion transport and resulting in undesired Li deposition. The Li-ion migration number of OICSEs can be obtained by the DC/AC electrochemical method proposed by Bruce [77, 78]. By assembling a Li |OICSEs| Li symmetric cell, an impedance spectrum test is performed before polarization begins, and then a minimal potential is applied for polarization tests, where the Li ions and anions move in opposite directions in response to an electric field. The lithium ions are reduced to Li atoms at the electrode interface, while the anions accumulated at the interface do not participate in the electrochemical reaction. Meanwhile, the anions can diffuse to the low potential electrode under concentration polarization. Finally, the impedance of the symmetric cell is tested after polarization. Based on the impedance change and current response, the Li^+^ migration number can be obtained using Eq. (3):

3 $$t_{{Li^{ + } }} = \frac{{I_{s} \left( {\Delta {\text{V}} - I_{0} R_{0} } \right)}}{{I_{0} \left( {\Delta {\text{V}} - I_{s} R_{s} } \right)}}$$

where $\Delta \text{V}$ is the dc polarization voltage, ${I}_{0}$ and ${I}_{s}$ are the initial and steady-state current, respectively. The ${R}_{0}$ and ${R}_{s}$ are the initial and steady-state interfacial impedance, respectively. Most OSEs are multi-ion conductors, so the Li-ion transference number of OSEs is generally low, usually only about 0.1–0.2 [79]. In contrast, ISEs are typically single Li-ion conductors with a migration number roughly equal to 1. Therefore, the ion migration number of composite ion conductors is generally more significant than that of OSEs.

### Electrochemical Stability

The electrochemical window is a vital parameter in evaluating the electrochemical stability of solid-state electrolytes. It determines the range of feasible reversible electrochemical reactions, facilitating controlled electrode potential during electrochemical desorption and adsorption processes and preventing irreversible reactions. The electrochemical window is typically measured by cyclic voltammetry or linear scanning voltammetry, using electrochemical cells containing working and reference electrodes for the configuration. The electrochemical window directly affects the lifetime and performance of the cell. Expanding the electrochemical window can enhance the compatibility of the solid-state electrolyte with both positive and negative electrodes, reduce energy losses and electrolyte degradation, and improve battery capacity retention and cycling stability. Generally, OICSEs offer a wider electrochemical window compared to OSEs. This phenomenon arises from the propensity of the polymer matrix in OSEs to decompose at high voltages, limiting their electrochemical window [80]. The most common oxidize potential of PEO-based polymer electrolytes is about 3.8 V, limiting their application in high energy density battery systems. Zhang et al. [81] developed an anion-immobilized OICSE to protect Li metal anodes by adding 40 wt% LLZTO to PEO (LiTFSI) polymer matrix. Compared to conventional liquid electrolytes with mobile anions, inorganic fillers effectively immobilize anions, resulting in uniform ion distribution and no dendritic lithium deposition. The wide electrochemical window (5.5 V vs. Li^+^/Li) of OICSE without distinct reaction was measured by LSV using Li|OICSE|SS. This indicates that OICSE has good polarization tolerance and great potential for high-voltage lithium batteries. The improvement of the OICSE electrochemical window is due to the excellent stability of LLZTO and its surface passivation layer towards lithium metal, while finely dispersed ceramic fillers help to remove impurities at the interface. Ding et al.[82] reported the addition of boron nitride (BN) to the PEO-LiTFSI system, BN reduces the crystallinity of PEO, promotes the dissociation of LiTFSI, and improves the ability of the PEO chain segment to transport ions, and the electrochemical stabilization window is increased from 4.43 to 5.16 V versus Li^+^/Li based on Li|OICSE|SS cell. The improvement in the electrochemical window is due to stronger binding between TFSI^−1^ and BN, which inhibits TFSI^−1^ transport and promotes Li^+^ transport. This slows down the concentration gradient and polarization and improves the stability of the lithium electrodeposition. Zhang et al. [83] prepared a flexible PEO/PEG-3LGPS composite electrolyte through an in situ coupling reaction, in which the ceramic and polymer were tightly bound to each other by strong chemical bonding, and successfully solved the interfacial compatibility problem. The oxidation potential of this PEO/PEG-3LGPS composite electrolyte was increased to 5.1 V versus Li^+^/Li based on Li|OICSE|SS cell. The enhancement of the electrochemical window was attributed to the higher ionic conductivity reducing the Li^+^ accumulation at the electrode/electrolyte interface, thus lowering the interfacial over-potential, and ultimately achieving better electrode–electrolyte compatibility.

### Electronic Conductivity

Electronic conductivity is often considered another key criterion for ASSLBs applications. Ideally, the electronic conductivity of a composite electrolyte should be as close to zero as possible, typically in the range of 10^−10^ S cm^−1^ or less. A recent study shows that the high electronic conductivity of solid electrolytes allows Li^+^ to combine with electrons to form lithium dendrites directly inside these SEs when the potential reaches the Li plating potential. Wang et al. [84] investigated the formation mechanism of dendritic grains in LLZO and Li_3_PS_4_ using operational neutron depth profiling (NDP), emphasizing the important role of reducing the electronic conductivity of SEs to achieve dendrite-free lithium plating at high current densities. Polymers typically have lower electronic conductivity (10^−14^ and 10^−17^ S cm^−1^) compared to inorganic materials. Therefore, the reduction of the electronic conductivity of electrolytes is favored by inorganic–organic composites. Goodenough et al. reported that CPE-25LZP has a low electronic conductivity of 9.0 × 10^–10^ S cm^−1^ at 25 °C [85]. Low electronic conductivity ensures that the electrolyte conducts ions rather than electrons, which avoids self-discharge and internal short-circuit problems in batteries.

### Thermal Stability

High thermal stability prevents OICSEs from decomposing during the thermal runaway of the battery, which plays a critical role in the safety of ASSLBs. Currently, thermogravimetric analysis (TGA) and differential scanning calorimetry (DSC) are commonly used techniques to analyze these properties. The thermal decomposition temperature and mass loss of the composite electrolyte can be measured by TGA, while the thermal stability and phase transition temperature of the material can be analyzed by DSC. Most inorganic electrolytes have high decomposition temperatures, so the addition of inorganic materials to OICSEs can improve the thermal stability of the electrolytes. For example, Ramaswamy et al. [86] investigated the thermogravimetric analysis curves of PVDF-HFP/POEGMA/LLZTO composite electrolytes by TG. The results showed that the weight of PVDF-HFP/POEGMA gradually decreased by about 25% from 240 to 395 °C for the membrane without LLZTO, while the weight of PVDF-HFP/POEGMA/LLZTO only gradually decreased from 245 to 420 °C, indicating that the incorporation of LLZTO filler improved the thermal stability. Meanwhile, the thermal stability of ASSLBs can be improved by introducing inorganic fillers. Cui et al. [87] reported a poly(propylene carbonate) (PPC) and 5 wt% LLZTO CSE. Commercial lithium-ion batteries using organic liquid electrolytes typically suffer severe performance degradation when operating temperatures exceed 60 °C. The solid-state battery of LiFePO_4_||Li based on the OICSE was operated at 160 °C with excellent rate capability at high rates, indicating that the OICSE can be used in the field of high-temperature lithium batteries. The melting point (T_m_), glass transition temperature (T_g_), and crystallinity (X_c_) can be obtained by DSC testing. The effect of SN plasticizer on the thermal properties of the PEO-LLZTO composite electrolyte was investigated [88]. The T_g_, T_m_, and X_c_ of the composite electrolyte gradually decreased with the addition of SN, when the content of SN was increased to 60 wt%, and the composite electrolyte with the highest ionic conductivity was obtained. Although plasticizers can improve the ionic conductivity of OICSEs by reducing polymer crystallization, they can also reduce the mechanical strength and the safety of ASSLBs, requiring a comprehensive consideration of the amount used.

## Mechanism of Li-Ion Transport in OICSEs

Ionic conductivity is one of the most crucial properties of OICSEs, determining whether OICSEs apply to practical devices. Consequently, the design and development of OICSEs with high ionic conductivity is imperative. This objective necessitates an in-depth understanding of the lithium-ion transport mechanism, a fundamental aspect for advancing the efficacy of these electrolytes in technological applications. The structure of OICSEs is believed to contain three main components: inorganic fillers, polymers, and interfaces formed by the interaction of inorganic fillers with polymers. However, adequate technical knowledge is still lacking to probe these complex microscopic nanoscale interfaces directly. Currently, solid-state nuclear magnetic spectroscopy (NMR) is considered a practical technical approach for understanding the lithium-ion transport mechanisms in OICSEs.

**Fig. 3:**
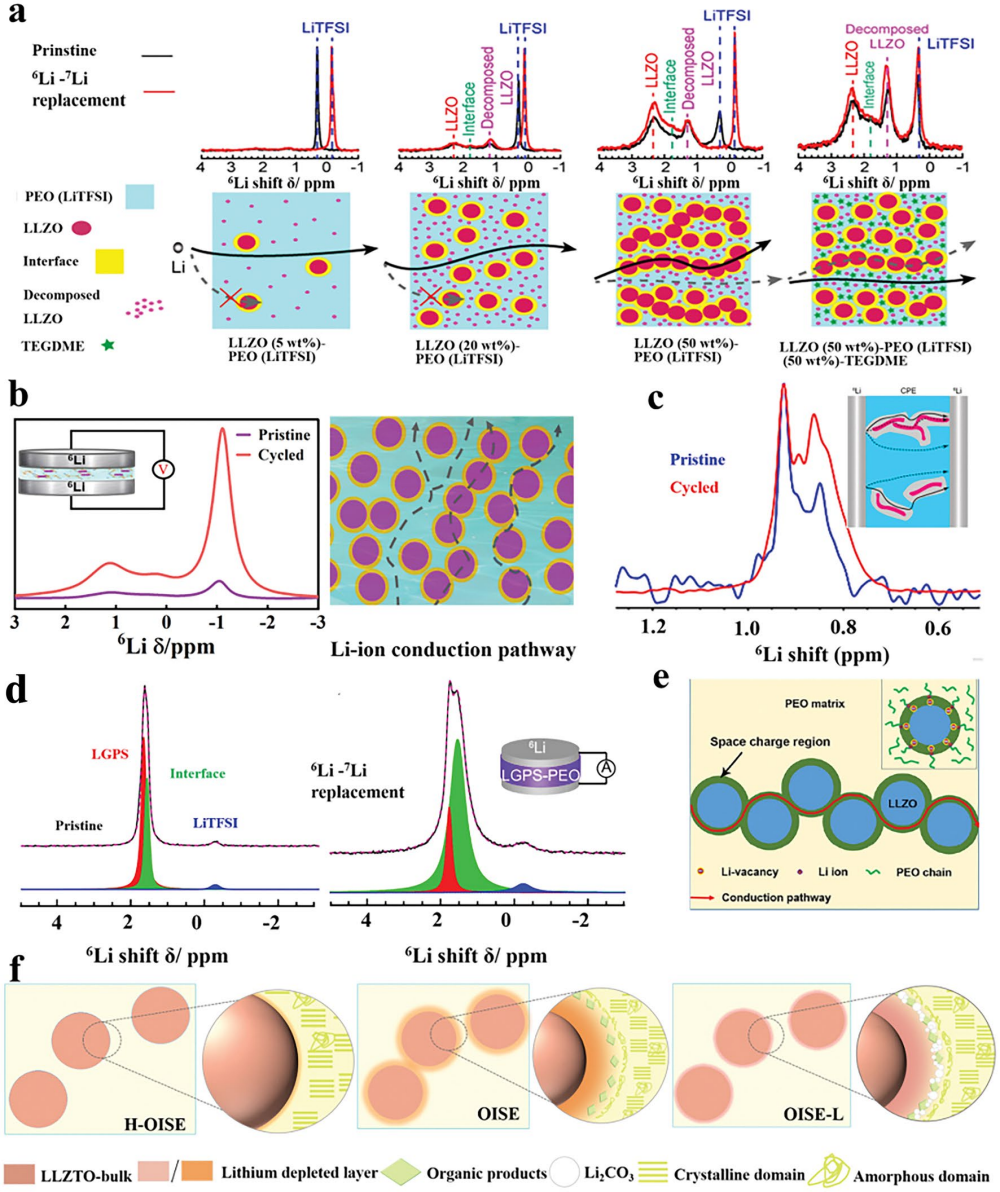
**a** ^6^Li NMR spectra of 5, 20, and 50 wt% LLZO-PEO/LiTFSI and 50 wt% LLZO-PEO/LiTFSI with TEGDME OICSEs before and after cycling and the corresponding Li-ion transport pathways [90], Copyright 2018, American Chemical Society. **b** ^6^Li NMR spectra of the LCPE-60 OICSEs before and after cycling and the Li-ion pathways [88], Copyright 2023 Elsevier. **c** ^6^Li NMR spectra of PAN (LiClO_4_)-5 wt% LLZO NWs OICSEs before and after cycling [92], Copyright 2017, American Chemical Society. **d** ^6^Li MAS NMR of an LGPS-PEO (LiTFSI) OICSE before and after cycling [93], Copyright 2019, American Chemical Society. **e** Schematic illustration of the ion conduction pathway along the space charge regions [94], Copyright 2018 American Chemical Society. **f** Schematic diagram of the interface of H-OISE, OISE, and OISE-L Copyright 2024 Wiley‐VCH GmbH [96]

Hu et al. first investigated the Li^+^ transport pathway in PEO (LiClO_4_/LLZO) OICSEs using the ^6^Li-^7^Li isotope tracing technique [89]. By assembling the ^6^Li/OICSEs/^6^Li system, the ^6^Li replaced the ^7^Li during the electrochemical cycling. Therefore, quantitative analysis of the resonance before and after isotope labeling can accurately quantify the contribution of different Li-containing components to ion conduction. These results indicated that ^6^Li in the LLZO increased by 39% after cycling. In contrast, the ^6^Li in the PEO phase and the interface were negligible, suggesting that the Li-ions prefer to go through LLZO rather than the PEO or PEO/LLZO interface. Subsequently, they further systematically investigated the effect of LLZO content on the ion conduction mechanism (Fig. 3a) [90]. When the LLZO content was below equal to 20 wt%, Li ions were mainly conducted through the PEO matrix. However, when the LLZO content exceeds a critical point, i.e., the LLZO particle forms a permeation network, which blocks the Li-ion conduction channel in PEO, leading to a transition of Li transport from the PEO phase to the LLZO. The specific transition point depends on various factors, such as the inorganic fillers' size, morphology, and composition. Furthermore, incorporating plasticizers into OICSEs results in the ion transport pathway reorientation, favoring the polymer phase [91]. Our recent work further demonstrates that in OICSEs containing plasticizer (SN), Li ions are mainly transported through the polymer phase, with LLZTO and the interface acting as synergistic conductors (Fig. 3b) [88]. This is attributed to the plasticizer reducing the polymer crystallinity and increasing the amorphous region, which is more conducive to lithium-ion transport.

The interfacial region formed by the interaction of inorganic fillers and polymers plays an important role in OICSEs. However, the conduction mechanism of the interfacial region is very complex, and it largely depends on the specific composition and structure of OICSEs. Yang et al. investigated OICSEs containing 5.0 wt% LLZO nanowires combined with PAN using the^6^Li NMR technique (Fig. 3c) [92]. The results showed that the ^6^Li ions in the PAN at 0.9 ppm remained unchanged after cycling, while the amount of ^6^Li in the PAN region modified by LLZO (0.85 ppm) was greatly enhanced. This indicates that Li ions prefer to be transported through the PAN region modified by LLZO (usually understood as the interfacial region) rather than the unmodified PAN phase. Zheng et al. reported OICSEs with different LGPS and Li salt contents using the ball milling method** (**Fig. 3d) [93]. The results showed that the largest interface in PEO(LiTFSI)-(EO/Li = 9:1)-70 wt% LGPS, while the ionic conductivity of OICSEs was positively correlated with the LGPS-PEO interfaces quantified by ^6^Li NMR spectrum. Therefore, PEO (LiTFSI)- (EO/Li = 9:1)-70 wt% LGPS electrolytes have stronger Li ions transport and more stable long-term cycling performance with lithium metal. The ^6^Li NMR tracer exchange technique shows that Li ions are mainly transported through the LGPS/PEO interface. The result further demonstrates the interface plays a significant role in ion conduction. In addition to the interface detected by the ^6^Li NMR spectrum, Li et al. observed the 3 nm space charge region between Ga-LLZO and PEO with transmission electron microscopy (TEM) [94]. The Li ions in the Ga-LLZO lattice move towards the surface, resulting in vacancies that are positively charged on the surface and negatively charged inside. When the space charge region on the surface of the nanoparticles is connected (Fig. 3e), the results show that the space charge region at the interface is a fast conduction pathway for Li ions. Based on both computational and experimental results, similar behavior was also found in the LATP/PEO OICSE [95]. The LATP in PEO can establish low-energy barrier hopping channels along the surface for lithium-ion migration. In general, the mechanism of ion conduction within OICSEs is complex, and whether the lithium ions are transported through the polymer phase, the bulk phase, or the interface depends on several factors, such as the type and structural composition of the OICSE, including the inorganic fillers content, size, and morphology. In addition, the ability of the interface to be a phase with fast lithium ions conductivity depends on the interfacial interactions between the organic and inorganic materials. Guo et al.[54] investigated that under the coexistence of DMSO and LLZTO, the coupling of DMSO molecules with LLZTO resulted in the redistribution of the electron density of the DMSO molecules, which induced aggregation of the charges around the sulfinyl group, thereby increasing the Lewis basicity of the sulfinyl group and enhancing the interaction between LLZTO filler and PAN matrix. This enhancement facilitates the uniform encapsulation of the polymer on the particles surface and the formation of continuous Li^+^ conduction channels between the ceramic and polymer, which induces dehydrocyanation of the PAN matrix. The LLZTO@PAN electrolyte shows sufficient ionic conductivity of 1.1 × 10^−4^ S cm^−1^, and a high Li^+^ transference number of 0.66. The Li|LLZTO@PAN/PEO|LFP cell delivers a high reversible capacity of 167 mAh g^−1^ at 0.1 C, as well as a small polarization of 0.06 V. Therefore, it is beneficial to improve the ionic conductivity of OICSE by constructing a continuous micro interface of composite electrolyte. In recent research, the mechanism of microscopic interface formation in composite electrolytes and the ionic conductivity mechanism has been investigated using 1D ^6^Li and 2D ^6^Li-^6^Li exchange NMR techniques (Fig. 3f) [96]. The interface signals in the ^6^Li NMR spectra are from the lithium-deficient layer of LLZTO. At high current densities, Li ions are conducted through the polymer phase, and the lithium-deficient layer, as well as LLZTO, play a synergistic role in promoting ionic conduction, but the Li_2_CO_3_ on the surface of LLZTO inhibits the transport of the lithium-deficient layer as well as LLZTO.

To compare the pathways of Li-ion conduction more clearly in different OICSEs systems, Table 1 summarizes the ion conduction pathways based on polymer, filler type, content, and the presence or absence of plasticizer. It is shown that the ion conduction pathway of the OICSE is highly dependent on the filler content, polymer system, plasticizer, and circulating current density, but one certain thing is that the micro interface plays an important role in the ion conduction of the OICSE.

**Table 1:** Possible Li-ion conduction pathways in different OICSEs systems

Inorganic filler	Polymer	Filler content (wt%)	Note	Li^+^ conduction pathway
LLZO	PEO	5		Polymer
LLZO	PEO	20		Polymer
LLZO	PEO	50		LLZO
LLZO	PEO	50	50 wt% PEGDME	Polymer
LLZTO	PEO	15	60 wt% SN	Mainly polymer, LLZTO and interface synergy to promote Li-ion conduction
LLZO	PAN	5		Interface
LAGP	PEO	70		Interface
LATP	PEO	10		Interface
LLZTO	PAN	94.3	DMSO	Interface
LLZTO	PEO	15	At high current	Mainly polymer, LLZTO and interface synergy to promote Li-ion conduction
LLZTO	PEO	15	At low current	Polymer, LLZTO and interface three-phase synergy to promote Li-ion conduction

## Key Inorganic Fillers and Advanced Structures in OICSEs

OICSEs are composed of polymers, lithium salts, and inorganic fillers. In 1973, Wright et al. [97] proposed that mixing alkali metal salts with PEO can conduct Li ions. Currently, polymer matrices include PEO [98], copolyvinylidene fluoride-hexafluoropropylene (PVDF-HFP) [99], polyvinylidene fluoride (PVDF) [100], polyethylene glycol diacrylate (PEGDA) [101], polymethyl methacrylate (PMMA) [102], polyvinyl carbonate (PVC) [103], tetramethyleneglycol methacrylate (TEGDMA) [104], and polystyrene (PS). These polymers are primarily semi-crystalline at RT, which limits chain segment movement, leading to low ionic conductivity (10^−6^ to 10^−8^ S cm^−1^) [105]. When the temperature is above the glass transition temperature, these polymers are in the amorphous region, and the ionic conductivity increases significantly. Lithium salts are generally classified as inorganic lithium salts and organic lithium salts. Inorganic lithium salts such as lithium perchlorate (LiClO_4_), lithium tetrafluoroborate (LiBF_4_), lithium hexafluoroarsenate (LiAsF_6_) [106], and lithium hexafluorophosphate (LiPF_6_), while inorganic lithium salts are organic compounds consisting of an electron-absorbing group added to the anion. Common organic lithium salts include lithium borate dioxalate (LiBOB), lithium difluoroxalate borate (LiDFOB), lithium bis(difluorosulfonyl)imide (LiFSI), and lithium bis(trifluoromethylsulfonyl)imide (LiTFSI), which are highly solubility in polymers and quickly form stable SEI films. The inorganic fillers can be divided into inert fillers and active fillers depending on whether they can conduct Li ions. Inert fillers are not involved in the conductive process and include ZnO, TiO_2_, SiO_2_, ZrO_2_, MgO, Al_2_O_3_, Y_2_O_3_, LiAlO_2_, BaTiO_3_, etc. [107, 108]; active fillers include garnet, chalcocite, NASICON, LISICON, perovskite, sulfide, Li_3_N, etc. Both inert and active fillers are regarded as plasticizers to reduce crystallization and promote the movement of Li ions. The inorganic fillers are available in various shapes such as nanoparticles (0D), one-dimensional (1D) nanofibers, nanorods, two-dimensional (2D) nanosheets, and three-dimensional (3D) frameworks. The inorganic fillers with different shapes can provide long-range permeation networks through the arrangement to promote Li ions conduction and increase the diffusion rate, thus forming a fast Li ions conduction pathway.

### Polymer with Inert Fillers

#### 0-Dimensional Inert Fillers

**Fig. 4:**
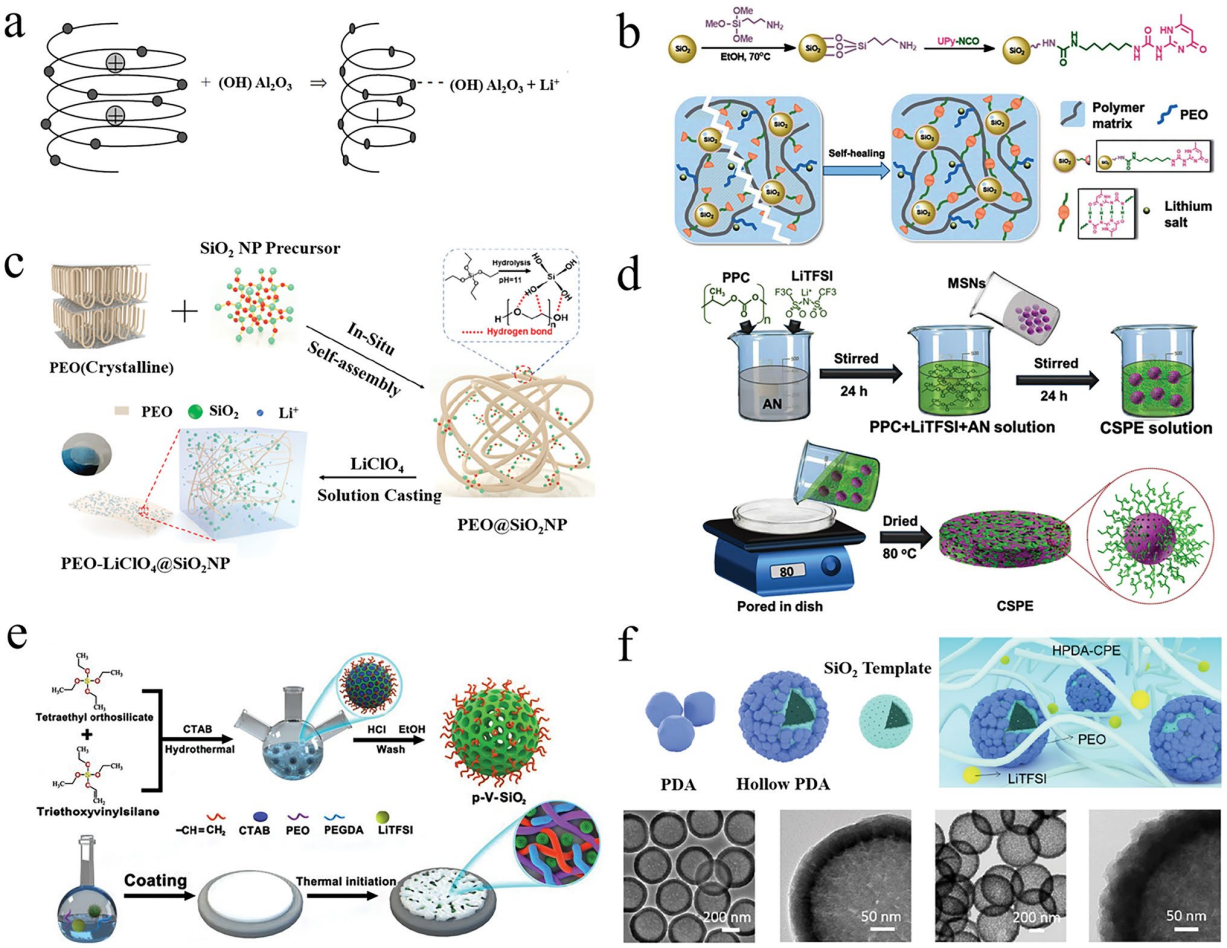
**a** Schematic diagram of the interaction between PEO chains and Al_2_O_3_ surface groups [109], Copyright 2004, Kluwer Academic Publishers. **b** Preparation process of SiO_2_-UPy and schematic diagram of SHCPE with supermolecule network structure [110], Copyright 2019 Royal Society of Chemistry. **c** Morphology and synthesis diagram of the PEO-LiClO_4_-SiO_2_ OICSEs [111], Copyright 2020 American Chemical Society. **d** Preparation process diagram of p–V–SiO_2_/PEO cross-linked OICSEs [113], Copyright 2021 Elsevier. **e** Synthetic routes of the PAN- insitu*-*SiO_2_ OICSEs [114]. **f** Preparation process diagram of hollow PDA composite nanospheres and the TEM images of hollow SiO_2_ and hollow PDA composites [115], Copyright 2022, American Chemical Society

0-dimensional (0D) inert materials are typically small filler particles with sizes ranging from a few nanometers to a few micrometers. These particles are introduced into polymer electrolytes with lithium salts to improve their mechanical properties, ionic conductivity, and electrochemical stability. This improvement is usually attributed to the inert fillers inhibiting the polymer crystallization, thus improving the chain segment motility. In addition, the Lewis acid–base interactions between groups on the nanoparticle surface and PEO chain segments, which can also facilitate the dissociation of lithium salts, have attracted extensive research. Croce et al. demonstrated that the improved electrochemical properties of PEO-based OICSEs were attributed to the –OH groups on the Al_2_O_3_ surface dispersed in the polymer matrix through the anionic “hydrogen bonding-mediated” solvation to reduce lithium salt association, thereby facilitating specific interactions between the filler, the polymer chain, and the ions from salt dissociation (Fig. 4a) [109]. Therefore, this objective can be achieved by incorporating more acidic sites, changing surface properties, or introducing functionalized nanomaterials. These strategies effectively inhibit polymer crystallization and enhance Lewis acid–base interactions between fillers, lithium salts, and polymer chains.

Xue et al. prepared an OICSE with a self-healing function by incorporating ureidopyrimidinone (UPy)-functionalized SiO_2_ into a polymer matrix containing UPy units (SiO_2_-UPy) [110], as shown in Fig. 4b. The OICSE shows a high ionic conductivity of 8.0 × 10^−5^ S cm^−1^ at 30 °C compared with that of the CPE blended with pristine SiO_2_. The improved ionic conductivity is attributed to the SiO_2_-UPy filler being uniformly dispersed in the polymer matrix through PEG-UPy hydrogen bonding. This increases polymer activity and the number of physical cross-linking sites in the matrix, enhancing the interaction with PEG-UPy. Yang et al. constructed PEO@SiO_2_ OICSEs with a 3D network structure of PEO and SiO_2_ particles by in situ assembly (Fig. 4c). The fusion of monodisperse SiO_2_ nanoparticles with 3D PEO successfully reduced the PEO crystallinity under the synergistic effect of strong Lewis acid–base and weak hydrogen bonding, achieving a high ionic conductivity of 1.1 × 10^−4^ S cm^−1^, and wide electrochemical window of 4.8 V. vs Li/Li^+^[111]. In addition, the construction method significantly promoted the stability of the solid electrolyte interface. Similar research results were published in PAN-based systems [112]. An interconnected fast Li^+^ conducting network was constructed by in situ hydrolysis of tetraethoxysilane (TEOS) within a polyacrylonitrile (PAN) matrix. This situ-formed interconnected inorganic network provides a robust backbone for the OSE and a sufficiently continuous surface with Lewis acidic sites, which will facilitate the dissociation of Li salts. As a result, the OICSE obtained a promising ionic conductivity of 3.5 × 10^−4^ S cm^−1^ and an attractive Young modulus of 8.627 GPa. When paired with a high-voltage cathode of LiNi_0.6_Mn_0.2_Co_0.2_O_2_, the ASSLBs exhibited a stable discharge capacity of 173.1 mAh g^−1^ with 93.8% retention after 200 cycles at 3.0–4.3 V. Nanofillers with high specific surface area help increase the interaction between the inorganic filler and the polymer matrix, increase the free volume of the polymer. Park et al. introduced highly mesoporous silica nanoparticles (MSNs) into bulk polypropylene carbonate (PPC) matrices with bendability and high stability (Fig. 4d) [113]. OICSEs have an ultra-high lithium-ion transference number of 0.86 due to the strong Lewis acid sites on the surface of the highly mesoporous MSNs, which enhance the interaction with the polymer matrix, form a homogeneous Li-ion transport phase between the polymer matrix and ceramic fillers, and improve the Li^+^ transference number ion mobility number of the OICSEs.

In addition to the different Lewis acid–base interactions caused by the surface chemistry of the filler particles, there is another important way to improve the polarity and the dispersion of the filler in the polymer matrix by modifying the nanofiller surface. Zhan et al. reported OICSEs with porous vinyl-functionalized silicon (p–V–SiO_2_) nanoparticles as fillers for PEO electrolytes with polyethylene glycol diacrylate (PEGDA) as a cross-linking agent (Fig. 4e) [114]. 10% p–V–SiO_2_/PEO OICSE exhibited the highest ionic conductivity of 5.08 × 10^−4^ S cm^−1^ at 60 °C, and a wide electrochemical stability window of 5.2 V vs. Li/Li^+^ based on Li|10% p–V–SiO_2_/PEO OICSE|SS cell. The improved electrochemical performance is attributed to well-interfacial compatibility between organic and inorganic materials due to cross-linking polymerization reactions between porous SiO_2_ and PEGDA in the PEO host, which promotes more lithium salt dissolution. Li et al. showed that the polydopamine (PDA) coated hollow silica nanoparticles were compatible with PEO and had a large interfacial contact area, as shown in Fig. 4f [115]. The thin polydopamine layer improved the compatibility with the polymer matrix and provided an effective and stable ion transport channel. Theoretical calculations show strong adsorption between polydopamine and TFSI^−^, which can inhibit the movement of TFSI^−^ anions. Compared with hollow SiO_2_ without PDA coating, this assembled PEO@PDA-SiO_2_ material exhibits higher ionic conductivity (1.89 × 10^−4^ S m^−1^), a wide electrochemical window (5.33 V vs. Li/Li^+^), and good mechanical strength. In addition, OICSE delivers a reversible capacity of 134.9 mAh g^−1^ after 205 cycles in comparison to 127.0 mAh g^−1^ for the undoped electrolyte. The dispersion of inorganic fillers in polymers can also be improved by certain technical methods; for example, Xie et al. used atomic layer deposition (ALD) to uniformly distribute ZnO quantum dots within a PEO-based solid electrolyte matrix. This method achieved a strong chemical interaction between VPI–ZnO and PEO and a uniform distribution of VPI–ZnO in PEO [116]. The results show that the loose O–Li^+^ coordination on the top surface of the electrolyte and the remaining VPI–ZnO lead to a significant increase in the Li^+^ migration number and a decrease in the interfacial resistance to Li metal. Furthermore, the NCM811|Li half-cell with the VPI–ZnO/PEO/LiTFSI exhibits a high discharge capacity of 164.7 mAh g^−1^ at 50 °C and has stable cycling performance.

Besides 0-dimensional inert oxides, ferroelectric materials can be incorporated into the polymer matrix as 0-dimensional inert materials, such as PbTiO_3_, BaTiO_3_, and SrBi_4_Ti_4_O_15_ [117, 118]. The ferroelectric materials exhibit strong Lewis acid–base characteristics, which can increase the polarity of polymer chains and further enhance the ionic conductivity in the interface region. Table 2 shows typical examples of 0D inert metal oxides and ferroelectric-filled materials in OICSEs.

**Table 2:** Properties of OICSEs with 0-dimensional inert fillers

Dimensional	Fillers	OICSEs	δ (S cm^−1^)	${T}_{{Li}^{+}}$	EW (V)	References
0D	SiO_2_	PEG-LiCF_3_SO_3_-13 wt% SiO_2_	4.8 × 10^−5^(40 °C)			[119]
0D	TiO_2_/Al_2_O_3_	PEO − 1 wt% TiO_2_/10 wt%Al_2_O_3_		0.6		[120]
0D	SiO_2_	PEO-LiN(CF_3_SO_3_)-5 wt% SiO_2_	1.4 × 10^−4^(RT)	0.2		[39]
0D	ZrO_2_	PVC-PMMA- − 10 wt% ZrO_2_	2.4 × 10^−5^ (30 °C)			[121]
0D	ZrO_2_	PEO_12_-LiClO_4_-7 wt% SO_4_^2−^/ZrO_2_	2.1 × 10^−5^ (RT)	0.287	4.95	[122]
0D	SrBi_4_Ti_4_O_15_	PEG: LiClO_4_-12.5 wt% SrBi_4_Ti_4_O_15_	2.432 × 10^−6^ (RT)			[123]
0D	ZrO_2_	P(EO)_20_LiClO_4_-5 wt% ZrO_2_			4.2	[124]
0D	ZrO_2_	P(EO)_20_(LiBF_4_)-10 wt% S-ZrO_2_		0.68		[107]
0D	TiO_2_/ZrO_2_	PVDF/PVC-2.5 wt%TiO_2_, PVDF/PVC-2.5 wt% ZrO_2_	5.43 × 10^−4^ (RT), 4.38 × 10^−4^ (RT)			[125]
0D	Y_2_O_3_	PEO-10 wt%Y_2_O_3_	5.95 × 10^−5^ (RT)			[126]
0D	BaTiO_3_	PEO-PVSDF-15 wt% BaTiO_3_	1.2 × 10^−4^ (RT)			[127]
0D	TiO_2_	PEO_20_-LiCF_3_SO_3_ − 10 wt% TiO_2_	10^−4^ (65 °C)	0.5		[40]
0D	PbTiO_3_	PVdF-HFP/LIBETI-(EC/DMC)-7.5%PbTiO_3_	4.18 × 10^−5^			[118]
0D	SiO_2_	PEO-MUSiO_2_	4.4 × 10^−5^ (30 °C)		5.5	[128]
0D	ZrO_2_	PMMA-SAN-6 wt% ZrO_2_	2.32 × 10^−4^ (RT)			[129]
0D	BaTiO_3_	PVDF-HFP/PVAC/7.5 wt% BaTiO_3_	2 × 10^−3^ S (RT)	0.48	5.4	[130]
0D	BaTiO_3_	PEO-LiTFSI-5 wt% BaTiO_3_	1.8 × 10^−5^ (25 °C)		4.7	[131]
0D	SiO_2_	PEO- LiClO_4_-15 wt%SiO_2_	6.31 × 10^−6^ (25 °C)		5.0	[132]
0D	BaTiO_3_	PEO-based-8 wt% BaTiO_3_	2.2 × 10^−5^ (25 °C)		4	[58]
0D	SiO_2_	SiO_2_-UPy	8.0 × 10^−5^ (30 °C)	0.39	5.1	[110]
0D	SiO_2_	PEO@SiO_2_	1.1 × 10^−4^ (30 °C)	0.367	4.8	[111]
0D	SiO_2_	PEO -PEGDA-SiO_2_	4.65 × 10^−3^ (RT)	0.45	5.4	[133]
0D	ZnO	VPI-ZnO/PEO/LiTFSI	1.5 × 10^−5^ (25 °C)	0.31	4.5	[116]
0D	SiO_2_	PPC-SiO_2_	8.5 × 10^−4^ (60 °C)	0.86	4.8	[113]
0D	SiO_2_	(PEO)_12_–6.85 wt%SiO_2_-LiClO_4_	3.03 × 10^−4^ (RT)			[134]
0D	SiO_2_	PEO-SiO_2_-PEGDA)	5.08 × 10^−4^ (60 °C)		5.2	[114]
0D	hollow PDA SiO_2_	PEO/LiTFSI/hollow PDA	1.89 × 10^−4^ (60 °C)	0.293	5.3	[115]
0D	SiO_2_	PAN-in situ-SiO_2_	3.5 × 10^−4^	0.52	5.2	[112]

#### 1-Dimensional Inert Fillers

One factor that improves the ionic conductivity of inert nanoparticles in OICSEs is the inhibition of the crystallization of polymers and an increase in the amorphous ratio. Another key factor is the suitable filler content that can provide a continuous percolation conduction pathway, thus significantly improving ionic conductivity. When the 0D inert filler concentration reaches a certain level in the polymer matrix, it leads to the accumulation of the particle filler, which severely affects lithium-ion conduction. Therefore, the 1D nanotube and nanofiber instead of 0D inert fillers are a reasonable choice to provide continuous percolation paths and improve the conductive behavior.

**Fig. 5:**
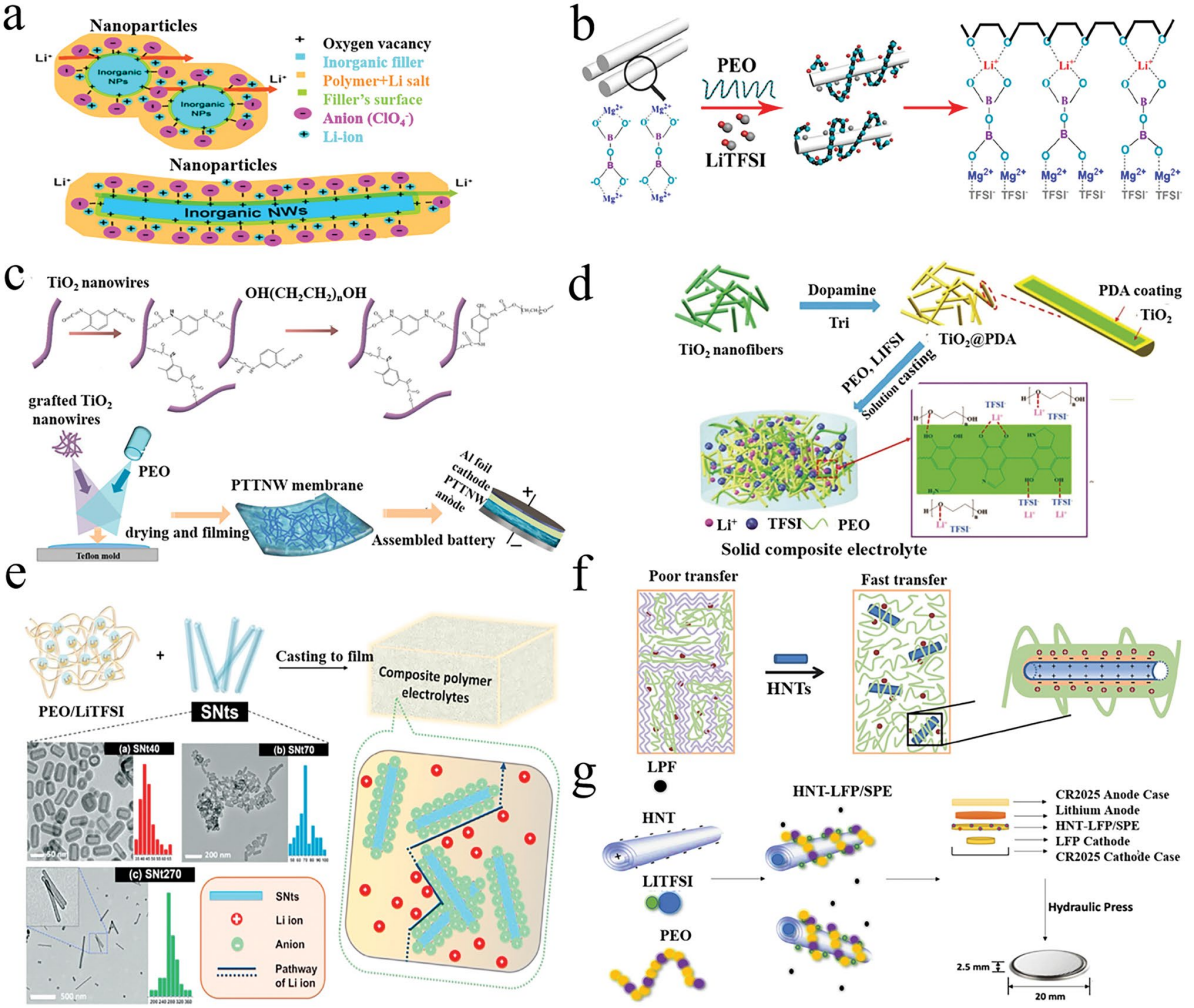
**a** Schematic illustration for Li-ion transport with nanoparticle and nanowire fillers [135], Copyright 2016, American Chemical Society. **b** Schematics of lithium-ion migration in Mg_2_B_2_O_5_ enhanced OICSEs [139], Copyright 2018, American Chemical Society. **c** Schematic diagram of TDI modified TiO_2_ and OICSE preparation [140], Copyright 2021 Elsevier. **d** Schematic diagram for the OICSEs fabrication procedure [141], Copyright 2022 Royal Society of Chemistry. **e** Schematic illustration depicting the formation of OICSEs incorporating silica nanotubes with hollow nanostructures [142] Copyright, 2020 Elsevier. **f** A mechanism to improve ionic conductivity by adding HNTs [143], Copyright 2018 Royal Society of Chemistry. **g** Schematic diagram of PEO-based HNTs electrolyte [144], Copyright 2019, American Chemical Society

Conventional 1D inert materials are mainly metal oxide nanowires, such as Y_2_O_3_ [135], TiO_2_ [136, 137], CeO_2_ [138], and Al_2_O_3_ [30]. Cui et al. reported a CSE containing Y_2_O_3_-doped ZrO_2_ (YSZ) nanowires with positively charged oxygen vacancies [135]. The results showed that the doped 7 mol% YSZ nanowires achieved the highest ionic conductivity of 1.07 × 10^−5^ S cm^−1^ at 30 °C, which is much higher than that of the electrolyte (2.98 × 10^−6^ S cm^−1^) containing 7% YSZ nanoparticles. The improved conductivity of the OICSE originates from the oxygen vacancies on the nanowire surface, which can act as Lewis acid sites to bind to the anions, as shown in Fig. 5a, effectively improving the ionic conductivity of the PAN-based OICSEs. Tao et al. reported PEO-based OICSEs containing 10% Mg_2_B_2_O_5_ nanowires, as shown in Fig. 5b [139]. The results showed that the ionic conductivity achieved 1.53 × 10^−4^ S cm^−1^ at 40 °C. This is attributed to the interaction of Mg^2+^ ions on the surface of Mg_2_B_2_O_5_ nanowires as Lewis acid centers with the anion TFSI^−^, thus weakening the interaction between Li^+^ and TFSI^−^, which in turn promoted the dissolution of the lithium salt and released more Li ions. In addition, the Mg_2_B_2_O_5_ nanowires have abundant Lewis acid sites [137], which enable the migration of Li ions in the two-phase interface between the electrolyte and Mg_2_B_2_O_5_ nanowires. TiO_2_ nanorod-filled polypropylene carbonate (PPC)-based OICSEs were prepared for the first time by Jing et al. The results indicate that the OICSE films with TiO_2_ nanorods can significantly improve the ionic conductivity (1.52 × 10^−4^ S cm^−1^) and have a stability electrochemical window (> 4.6 V vs. Li^+^/Li based on Li|OICSE|SS cell) and a tensile strength of 27 MPa at RT. This is attributed to the TiO_2_ nanorods providing more continuous lithium-ion transport channels and their surface porosity and composition improving the interfacial contact between polymer and filler and Lewis acid–base reaction sites.

Beyond integrating one-dimensional (1D) nanomaterials into the polymer matrix, the ionic conductivity can be further augmented through surface chemical modification. Li et al. successfully prepared a novel organic–inorganic cross-linked PEO-TDI-TiO_2_ electrolyte film using toluene-2,4-diisocyanate (TDI) as a modifier, as shown in Fig. 5c [140]. The OICSE membrane has a high ionic conductivity of 1 × 10^−4^ S cm^−1^ at 30 °C, and a high Li^+^ transference number of 0.36 at 60 °C. The wide electrochemical window (5.5 V vs. Li^+^/Li) was determined by LSV with the asymmetric battery of Li foil|OICSE| stainless steel (SS). The surface modification of TDI helps reduce the surface energy of TiO_2_ nanowires, thus enabling the polymer matrix chains to form effective covalent bonds with the nanofillers. Furthermore, the cross-linked and branched network structure effectively increases the amorphous regions in the polymer matrix. Zhao et al. reported a filler surface coating method, which involves coating a polydopamine (PDA) layer on the TiO_2_ nanofibers surface and then incorporating it into the PEO matrix (Fig. 5d) [141]. This coating method inhibited the filler aggregation in the PEO matrix and enhanced the compatibility between the PEO matrix and the PDA. The strong lithophilic layer of PDA also improved the ionic conductivity behavior at the filler/polymer interface, enabling the OICSEs to exhibit a high ionic conductivity of 4.36 × 10^−4^ S cm^−1^ and a wide electrochemical window of 5 V versus Li^+^/Li at 55 °C were studied by LSV using a Li|OICSE|SS cell. Xue et al. successfully synthesized a series of one-dimensional silica nanotubes (SNts) with hollow nanostructures and high uniformity by etching rod-shaped nickel hydrazine complexes for PEO-SNts (Fig. 5e) [142]. Compared with OICSEs based on 0D silica nanoparticles, PEO-SNts indicate significantly improved conductivity, thermal stability, and cycling stability.

Halloysite nanotubes (HNTs) are a unique natural 1D nanomaterial in addition to metal oxide 1D materials. It has the characteristics of tubular nanostructures, high aspect ratio, versatility, good biocompatibility, and high mechanical strength, and has received widespread attention in many fields. HNTs are a hydrated polycrystalline 1:1 layered silicate clay mineral with an outer siloxane surface and an inner alumina core. Therefore, the outer surface is generally negatively charged, like SiO_2_. Chen et al. prepared an OICSE-5 by introducing 5% natural halloysite nanotubes (HNTs) into polyvinylidene fluoride (PVDF). The ionic conductivity (3.5 × 10^−4^ S cm^−1^) was improved by ten orders of magnitude at 30 °C compared to the electrolyte without HNTs (Fig. 5f) [143]. The improvement in ionic conductivity was mainly attributed to the negatively charged outer surface and high specific surface area of HNTs, which facilitated the migration of Li ions in PVDF. However, the interfacial compatibility of HNT nanotubes with LFP electrodes is poor. To address this issue, Miller et al. reported a modification method in which a small amount of LFP was added during the preparation of OICSEs (Fig. 5g) [144]. This modification increased ionic conductivity, and the compatibility between electrolyte and electrode was significantly enhanced. Moreover, the electrochemical stability window was improved to 5.14 V, and the Li^+^ transference number was 0.46. The HNTLFP/SPE-based LFP polymer batteries present stable discharge capacities of 120 ± 3 mAh g^−1^ at 0.5 C after 300 discharge/charge cycles. In addition, metal–organic framework (MOF) nanorods and nickel phosphate (VSB) nanorods can also be introduced into the polymer matrix as effective 1D solid fillers to improve the electrochemical performance of OICSEs [145–148].

#### 2-Dimensional and 3-Dimensional Inert Fillers

Previous studies have demonstrated that inert nanoparticles and nanofibers can enhance ionic conductivity by suppressing the polymer crystallinity and providing continuous ionic conduction channels. To further enhance the ionic conductivity and improve the mechanical properties of OICSEs, researchers introduced 2D nanosheets and even developed 3D inorganic framework nanostructures. These structures provide continuous three-dimensional channels with no cross-connections between the inorganic phases. The thermal stability and mechanical properties were significantly improved by modulating the contact-specific surface area of the polymer with the filler. In recent works, 2D inert materials in OICSEs mainly include graphene oxide (GO), montmorillonite (MMT), boron nitride (BN), and MXenes nanomaterials. In contrast, 3D inorganic framework materials mainly cover metal oxides (e.g., Al_2_O_3_, SiO_2_, BaTiO_3_) and glass fibers. The typical examples of the electrochemical performance of OICSEs containing 2D nanosheet structures and 3D network frameworks are summarized in Table 3.

**Table 3:** Properties of OICSEs with 2-dimensional and 3-dimensional inert fillers

Type	Filler	OICSEs	δ (S cm^−1^)	T_*Li*_^+^	EW(V)	References
2D	LDHs	PEO/OMLDH	1.61 × 10^−5^ (20 °C)	0.42		[162]
2D	PGO	BCP-0.3 wt% PGO	2.1 × 10^−4^ (30 °C)			[163]
2D	GO	0.6 wt% GO)/LiClO_4_/PEO				[164]
2D	MXene Ti_3_C_2_T_x_	Ti_3_C_2_T_x_/PEO				[165]
2D	Kaolinite	PEO/20 wt%PK	6.1 × 10^−5^ (RT)			[166]
2D	GO	PAN-LiTFSI-0.9 wt% GO	1.1 × 10^−4^ (30 °C)	0.4	5	[167]
2D	BN	10 wt% hBN/PAN	1.0 × 10^−3^ (RT)		4.7	[168]
2D	MMT	(PEO-PMMA)- 10 wt% EC-3 wt% MMT	1.0 × 10^−5^ (RT)			[169]
2D	FGnP	PEO/LiClO_4_/FGnP(0.5%)	2.53 × 10^−5^			[170]
2D	GO	LiClO_4_-PAN-1wt% GO	4.0 × 10^−4^ (30 °C)		4.3	[171]
2D	Vermiculite sheets	PEO/LiTFSI/10% VS	2.9 × 10^−5^ (25 °C)	0.125	5.3	[43]
2D	g-C_3_N_4_	PEO/5% g-C_3_N_4_	1.7 × 10^−5^ (30 °C)	0.56	4.7	[156]
2D	BN	PVDF-HFP-1 wt% BN	1.82 × 10^− 3^(RT)		4.8	[154]
2D	Lepidolite	Lepidolite-PEO-LiClO_4_	1.6 × 10^−4^ (60 °C)			[172]
2D	MXene Ti_3_C_2_T_x_	PEO_20_-LiTFSI-3.6 wt% Ti_3_C_2_T_x_	2.2 × 10^−5^ (28 °C)	0.18	4.7	[173]
2D	MMT	PEC-LiMNT	3.5 × 10^−4^(25 °C)	0.83	4.6	[152]
2D	MMT	PEO/MMT	4.7 × 10^−3^(70 °C)		4.6	[174]
2D	GO	PEO -LiClO_4_-0.21 wt% GO	5.7 × 10^−5^ (RT)	0.47		[175]
2D	MXene-Ti_3_C_2_	MXene-2 wt% mSiO_2_/ePPO	4.6 × 10 ^4^ (RT)		4.3	[157]
2D	MnO_2_	PEO/LiTFSI/5% MnO_2_	1.95 × 10^−5^(30 °C)	0.378	4.5	[176]
2D	BN	12 wt% BN-PEO-PVDF	2 × 10^−4^ (70 °C)			[155]
2D	MOF	8% MOF/PEO/LiTFSI	1.66 × 10^−5^ (25 °C)	0.378	4.9	[177]
2D	LDH	PEO/5 wt% 2D LDH	2.7 × 10^−4^ (60 °C)	0.42	5	[178]
2D	MMT	LiTFSI/OMMT /CA/PVDF	3.40 × 10^−4^ (25 °C)	0.315	4.2	[179]
2D	BNN	BNNs-MPS-PEGDA	1.05 × 10 ^−4^ (RT)	0.49	5.5	[180]
2D	LiDGO	PEO/LiTFSI/LiDGO	3.4 × 10^−5^ (30 °C)	0.57		[150]
2D	MMT	MPEGA-PEGDA-3 wt%MMT	1.06 × 10^−3^ (RT)	0.79	5	[181]
2D	GO	1 wt% GO-PEO	1.54 × 10^−5^ (24 °C)	0.42	5	[149]
2D	MMT	GPE/VAMMT	1.08 × 10^−3^ (RT)	0.8	4.9	[153]
2D	HUT_4_	PEO-10%HUT_4_	5.3 × 10^−4^ (90 °C)	0.62	5.4	[182]
3D	CNT	PEO-10% clay-CNT	2.07 × 10^−5^(RT)			[71]
3D	Al_2_O_3_	PEO-Al_2_O_3_				[183]
3D	Al_2_O_3_	PEO-LiTFSI-Al_2_O_3_	5.82 × 10^−4^(RT)			[158]
3D	SiO_2_	SiO_2_-aerogel/PEO	0.6 × 10^−3^(30 °C)	0.38	4.4	[161]
3D	BaTiO_3_	BaTiO_3_-PEO-LiTFSI	5.83 × 10^−5^(30 °C)		5.8	[159]
3D	GF	PEO-SN_25_-LiTFSI10-GF	2.85 × 10^−4^(RT)		5.5	[184]
3D	GFC	PEO@GFC-25% ILs	1.6 × 10^−4^ (30 °C)	0.45	5.2	[160]
3D	SiO_2_	PEO-C-SiO_2_	1.9 × 10^−4^(30 °C)	0.3	5.4	[185]

**Fig. 6:**
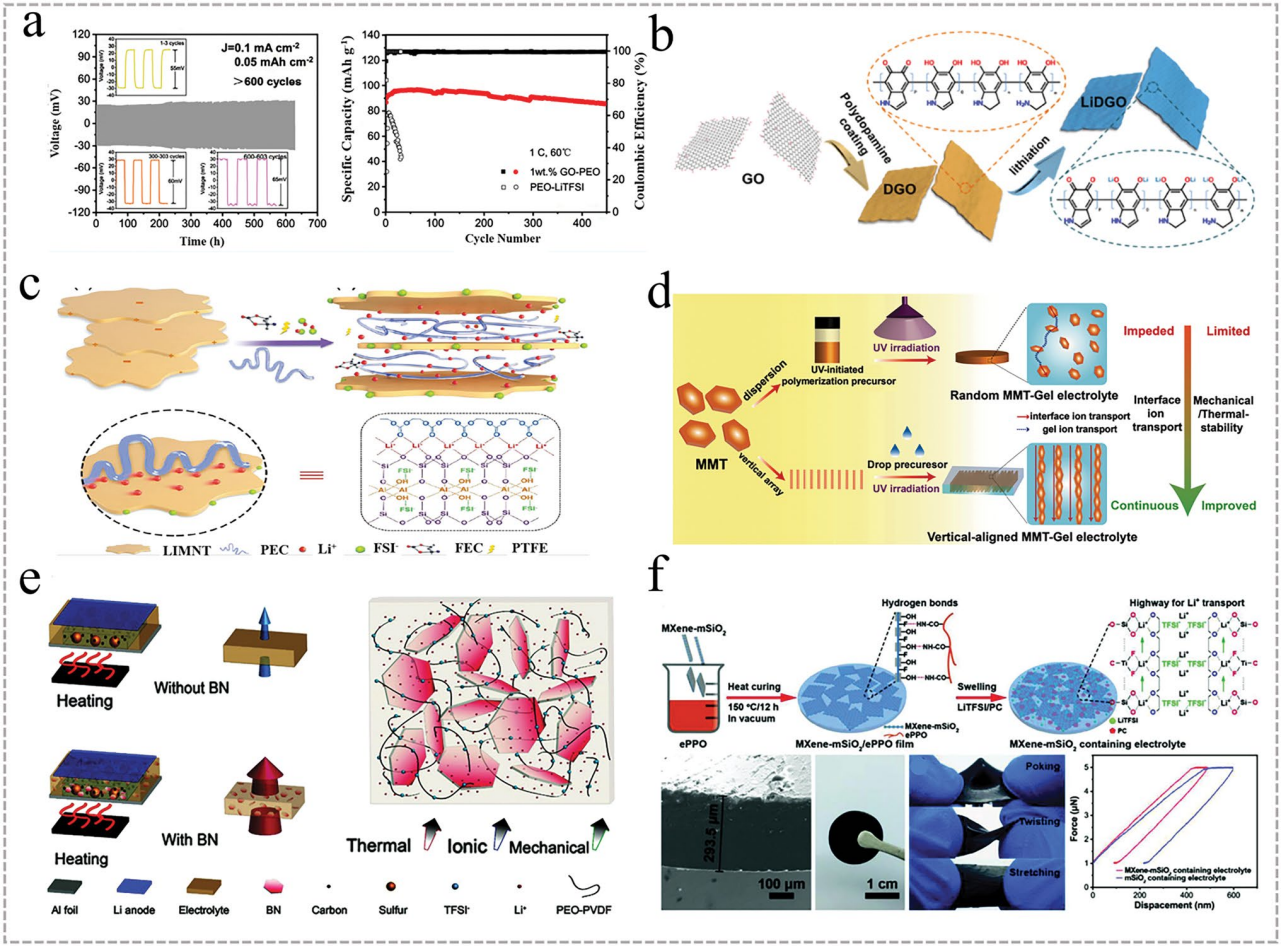
**a** Voltage–time profiles of Li||GO-PEO||Li at 60 °C and cyclic performance of full battery at 1C [149], Copyright 2021, American Chemical Society. **b** Schematic diagram of the preparation of LiDGO nanosheets [150], Copyright 2020 Elsevier. **c** Schematic diagram of ion migration mechanism of LiMNT interlayer insertion into PEC-based electrolyte [152], Copyright 2019 WILEY. **d** Schematic diagram of the manufacturing process of GPE/VAMMT [153], Copyright 2022 Xinyang Li. **e** Schematic diagram of heat transfer in electrolytes with and without BN additives [155], Copyright 2020 Guangyuan Wesley Zheng. **f** Schematic diagram of the manufacturing containing MXene mSiO_2_ [157], Copyright 2020 WILEY

GO is a graphene derivative with a two-dimensional layered structure that contains various hydrophilic functional groups such as –C–O–C, –CO, –COOH, and –OH on the surface, giving it excellent hydrophilicity and dispersibility. Xu et al. added 1 wt% of graphene oxide (GO) to PEO-based electrolytes for OICSEs and achieved an ionic conductivity of 1.54 × 10^−5^ S cm^−1^ at 24 °C [149], Li^+^ transference number of 0.42. The wide electrochemical window (about 5 V vs. Li^+^/Li) was measured by LSV using Li|GO-modified PEO|SS. The symmetric Li||GO-PEO||Li cell was stably cycled at an overpotential of 27 mV for 600 h, as shown in Fig. 6a. In addition, the LiFePO_4_//GO-PEO//Li cell exhibited excellent cycling, with a discharge capacity of 142 mAh g^−1^ at 0.5 C and 91% capacity retention after 100 cycles, indicating that it can inhibit the growth of lithium dendrites. The enhancement of ionic conductivity depends on the continuity of the conduction channels and the lithium-ion concentration. Thus, the ionic conductivity of OICSEs can be further enhanced by increasing the local lithium-ion concentration in the interfacial regions. Wu et al. synthesized lithiated polydopamine-modified graphene oxide nanosheets (LiDGO) and doped them into a PEO matrix, as shown in Fig. 6b [150]. A comprehensive evaluation of the electrochemical properties showed that the long-range conduction pathway with localized lithium-ion concentration constructed at the PEO/LiDGO interface significantly enhanced the ionic conductivity of OICSEs. The ionic conductivity reached 3.4 × 10^−5^ S cm^−1^ at 30 °C and had excellent mechanical stability. The full battery achieves a discharge capacity of ~ 156 mAh g^−1^ after 200 cycles with ultra-high-capacity retention of 98.7%. Xiong et al. introduced interatomic lithium montmorillonite (Li-MMT) into lithium-sulfur batteries for the first time and achieved free migration and exchange of interlayer cations in a thick sulfur cathode [151]. This work demonstrated that natural montmorillonite clay possesses a cation exchange function and can facilitate conduction by replacing other cations with Li ions. Zhang et al. prepared an OICSE consisting of poly(ethylene carbonate), layered lithium montmorillonite (LiMNT), and high-pressure fluorocarbon subethylenes (PEC) using a combination of solution casting and hot pressing [152]. The OICSE acquires a high ionic conductivity of 3.5 × 10^−4^ S cm^−1^ and a high Li^+^ transference number of 0.83 at 25 °C. A wide electrochemical window of 4.6 V versus Li^+^/Li was evaluated by LSV using Li foil|OICSE|SS. The mechanism of the enhanced Li^+^ transference number in OICSE is attributed to the selective immobilization of charged species. The upper and lower surfaces of the nanoflake LiMNT equipping –Si–O–Si- silicon tetrahedral sheets are negatively charged, and edge-shared faces consisting of –Al–OH groups are positively charged (Fig. 6c). When the PEC-Li polymer electrolyte is inserted into the intercalation of LiMNT, this surface difference allows selective immobilization of the charged material. The lithium salt anions are more likely to approach the edges of LiMNT, while the Li^+^ cations are more likely to be present in the intercalation space. Meanwhile, the carbonate group (–O–(C=O)–O–) with many lone pair electrons in the PEC will interact with the free Li^+^. This interaction leads to an ordered entry of Li^+^ into the interlayer space. As a result, this arrangement shortens the Li^+^ transport pathway and provides an efficient transport channel resulting in a high Li^+^ transfer number. To effectively solve the inhomogeneous ion transport problem and improve the thermal stability and mechanical properties of OICSEs. Ding et al. utilized a directional freezing method to prepare vertically aligned MMT arrays with ultra-low curvature (Fig. 6d) [153]. A uniform and continuous ion-conductive interface was formed in the OICSEs through UV-induced polymerization, facilitating Li^+^ migration. The results demonstrated that CSE/VAMMT exhibited higher Li-ion transference numbers and ionic conductivity at RT (1.08 mS cm^−1^). Moreover, it displayed excellent cycling stability, with no short-circuiting during continuous lithium deposition/stripping for 1000 h. The 2D BN nanosheets have attracted considerable attention due to the ability of the B atoms to interact with the anions of lithium salts as Lewis acid sites on the planar surface, thereby releasing more Li ions and enhancing ionic conductivity [154]. Zheng et al. developed a hybrid polymer electrolyte (BN-PEO-PVDF) containing 2D BN nanosheets, as shown in Fig. 6e [155]. In addition to improving ionic conductivity and mechanical properties, BN enhanced the thermal stability of the PEO-based electrolyte, allowing the BN-PEO-PVDF electrolyte to balance thermal changes faster and achieve more uniform ion transport. Ding et al. introduced g-C_3_N_4_ nanosheets similar to BN into PEO-based electrolytes, improving electrochemical performance, mechanical properties, and thermal stability [156]. Furthermore, MXene is a common 2-dimensional metal carbide layered material with a negative charge due to the surface with rich polar groups, such as –OH, –Cl, and –F. It has a strong interaction with lithium salts, which helps in the dissociation of lithium salts. Yang et al. incorporated insulating MXene-mSiO_2_ nanosheets into the PEO electrolyte, as shown in Fig. 6f [157]. Due to the abundant functional groups of MXene-mSiO_2_, the Lewis acid–base interactions between the PEO chain and anions were promoted, enabling the rapid transport of Li^+^ ions across the mesoporous nanosheet/polymer interface. As a result, the OICSE exhibited high ionic conductivity of 4.6 × 10^−4^ S cm^−1^ and Young's modulus of 10.5 MPa, Young's modulus is 34 orders of magnitude higher than that of the silica particle/ePPO electrolyte. Noteworthy, the full cell exhibits a long and stable cycle performance up to 250 cycles under 0.5 C at 25 °C, and the capacity is well maintained at 141.8 mAh g^−1^, much higher than that of the LFP cathodes with pure ePPO electrolyte (60.3 mAh g^−1^).

**Fig. 7:**
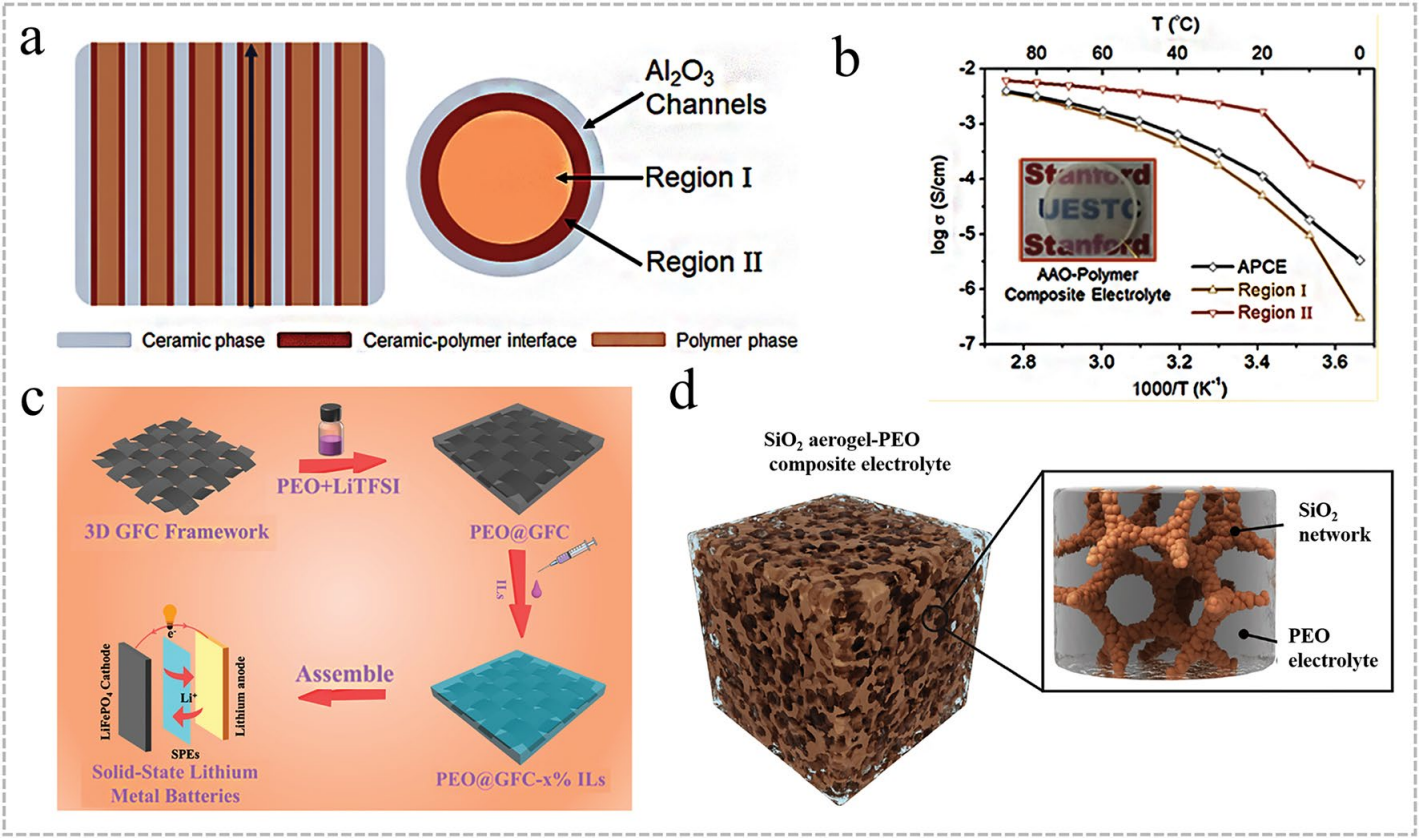
**a** Schematics of OICSEs with three types of geometrical structures. **b** Ionic conductivity in different regions of composite electrolytes [158], Copyright 2018, American Chemical Society. **c** Schematic illustration for preparation of PEO@GFC-25%ILs [160], Copyright 2020 Elsevier. **d** Schematic diagram of the microstructure of OICSEs containing 3D SiO_2_ aerogel [161], Copyright 2018 WILEY

In recent years, the ice template method, electrostatic spinning, sol–gel method, and 3D inorganic skeletons have been reported to construct continuous ion transport channels to form 3D OICSEs. The strategy of building OICSE with a three-dimensional skeleton structure solves the accumulation problem and further improves mechanical strength. Zhang et al. reported an OICSE with vertically aligned and continuous nanoscale ceramic-polymer interfaces using modified Al_2_O_3_ as the skeleton and PEO as the polymer matrix, as shown in Fig. 7a [158]. The Li^+^ transport along the ceramic/polymer interface was demonstrated for the first time, and the interfacial ionic conductivity was predicted to be higher than 10^−3^ S cm^−1^ at 0 °C, as shown in Fig. 7b. The ionic conductivity was 5.82 × 10^−4^ S cm^−1^ at RT, which was four orders of magnitude higher than that of the OICSE with nanoparticles and nanowires. The improvement of ionic conductivity is mainly attributed to the high aspect ratio of the polymer/ceramic interface formed by the vertically aligned 3D Al_2_O_3_ and the polymer matrix, which allows Li ions to conduct along the continuous vertically aligned interface and effectively reduces the crystallization of the polymer. Han et al. explored a simple and efficient solution-blowing technique to prepare well-aligned BaTiO_3_ nanofibers with an average diameter of about 300 nm combined with PEO polymers to form OICSEs [159]. Compared with the electrolyte without BaTiO_3_, the ionic conductivity increased from 5.74 × 10^−6^ to 5.83 × 10^−5^ S cm^−1^ at 30 °C, and the Li/Li^+^ electrochemical stability window was increased to about 5.8 V. To further enhance the ionic conductivity and electrochemical stability of OICSEs, Zhang et al. introduced an ionic liquid into a PEO-based 3D glass fiber cloth (PEO@GFC-25%ILs) framework (Fig. 7c) [160]. The results showed that PEO@GFC-25%ILs exhibited a high ionic conductivity of 1.6 × 10^−4^ S cm^1^ at RT, and an electrochemical window of 5.2 V versus Li^+^/Li was performed by assembling a Li|OICSE|SS cell. The Li|PEO@GFC-25%ILs|Li cells also demonstrated excellent cycling performance and rate capability with stable cycling of 2000 h. The full batteries assembled based on LCO and LFP cathode with PEO@GFC-25% ILs electrolyte can achieve specific capacities of 128.3 mAh g^−1^ and 155.2 mAh g^−1^, respectively. Furthermore, the LFP/PEO@GFC-25% ILs/Li battery can provide a reversible capacity of 152.0 mAh g^−1^ after 150 cycles at 0.5 C. To improve the mechanical properties of OICSEs to effectively suppress the occurrence of lithium dendrites and achieve high ionic conductivity at RT. Cui et al. synthesized a novel 3D SiO_2_ aerogel backbone by sol–gel method, injected with PEGDA, SN, and LiTFSI, and finally formed OICSEs by ultraviolet photocuring (Fig. 7d) [161]. This interconnected SiO_2_ aerogel strengthens the skeletal structure of all the OICSEs and offers a substantial and uninterrupted surface area for anion adsorption, creating a highly conductive pathway. As a result, the OICSEs achieve a high modulus of approximately 0.43 GPa and a remarkable ionic conductivity of 6 × 10^−4^ S cm ^−1^ at 30 °C.

Although the significant enhancement effect of aligned structures on ion conduction behavior has been demonstrated, the methods for preparing these structures still need to be more thoroughly explored. Some limitations and challenges may still exist in the current preparation methods, such as process complexity, material selection, and interface engineering. Further research can be devoted to the development of simpler, scalable preparation methods while optimizing material combinations and interfacial interactions for more efficient ion conduction and optimized electrochemical properties. In addition, the long-term stability, cycle life, and compatibility with electrodes of these aligned structures also need to be explored to ensure reliability and durability in practical applications. In summary, although the aligned structures have potential in ion conduction behavior, further research extensions are still needed to realize their practical applications.

Table 4 shows the advantages and disadvantages of inert materials with different dimensions, where the 0D inert materials have good mechanical properties and chemical stability, but low ionic conductivity and poor interfacial contact. 1D Nanowires/nanotubes are beneficial to some extent to improve interfacial contact and inhibit crystallization of polymers, especially when the orientation is consistent, and can provide continuous interfacial conduction for Li-ions, but the preparation process is complicated. The 2D inert materials with high specific surface area, good interfacial contact, and rich functional groups on the surface (e.g., MXene-Ti_3_C_2_ and BN, which contain functional groups such as –OH, –O, –NH_2_, and –F.) can interact with Li ions in the OICSEs and further promote Li-ion migration, but have poor mechanical properties. The 3D inert material has high mechanical strength, and thermal stability, which promotes the formation of a continuous conductive interface with the polymer and improves the ionic conductivity of the OICSEs, but the preparation method is complicated and requires special equipment.

**Table 4:** Advantages and disadvantages of inert materials in different dimensions

Inert fillers	Example	Advantage	Disadvantage
0D particles	SiO_2_/Al_2_O_3_/ZrO_2_	High mechanical strength and hardness Excellent chemical stability Good thermal stability	Low ionic conductivity Poor interfacial contact with electrolyte and electrode materials
1D nanotube/ nanofiber	HNTs TiO_2_/Y_2_O_3_ Nanowires	Relatively high specific surface area Excellent chemical stability Good mechanical properties	Low ionic conductivity Complex preparation process HNTs with brittle structure
2D Nanosheet	MXene-Ti_3_C_2_, MMT, GO, BN	High specific surface area Multifunctional surface functional groups (e.g., –OH, –NH_2_, –F, etc.) Good mechanical properties	Large differences in ionic conductivity (e.g., MXene-Ti_3_C_2_, GO) High preparation process and cost
3D Network	3D-Al_2_O_3_, 3D-SiO_2_	High specific surface area Excellent mechanical properties and thermal stability	Low ionic conductivity Complex preparation process

### Polymer with Active Fillers

Active fillers have high ionic conductivity and electrochemical activity relative to inert fillers, and they can participate in electrochemical reactions and provide additional ion transport channels, thereby improving the ionic conductivity and the electrochemical performance. Therefore, active materials are known as fast ion conductors and could provide a highly efficient pathway for Li-ion. However, the active fillers may lead to a certain degree of electrode polarization and capacity degradation, and their properties need to be optimized and regulated to improve the cycle life and stability of the battery. Typical active materials based on solid-state electrolytes consist of sulfide-type, garnet-type, NASICON-type, and perovskite-type materials.

#### Polymer Matrix Incorporating Sulfide-Type Materials

Sulfide electrolytes are characterized by substituting sulfur ions for oxygen ions, resulting in larger ion transport pathways for Li ions. As a result, they exhibit relatively high ionic conductivity, typically ranging from 10^−3^ to 10^−2^ S cm^−1^, comparable to liquid electrolytes. However, sulfide electrolytes have poor electrochemical stability and unstable interface contact with lithium metal, leading to decomposition reactions and high interfacial impedance [186]. Generally, sulfide electrolytes are combined with polymers or lithium alloys as anodes to improve interface stability.

**Fig. 8:**
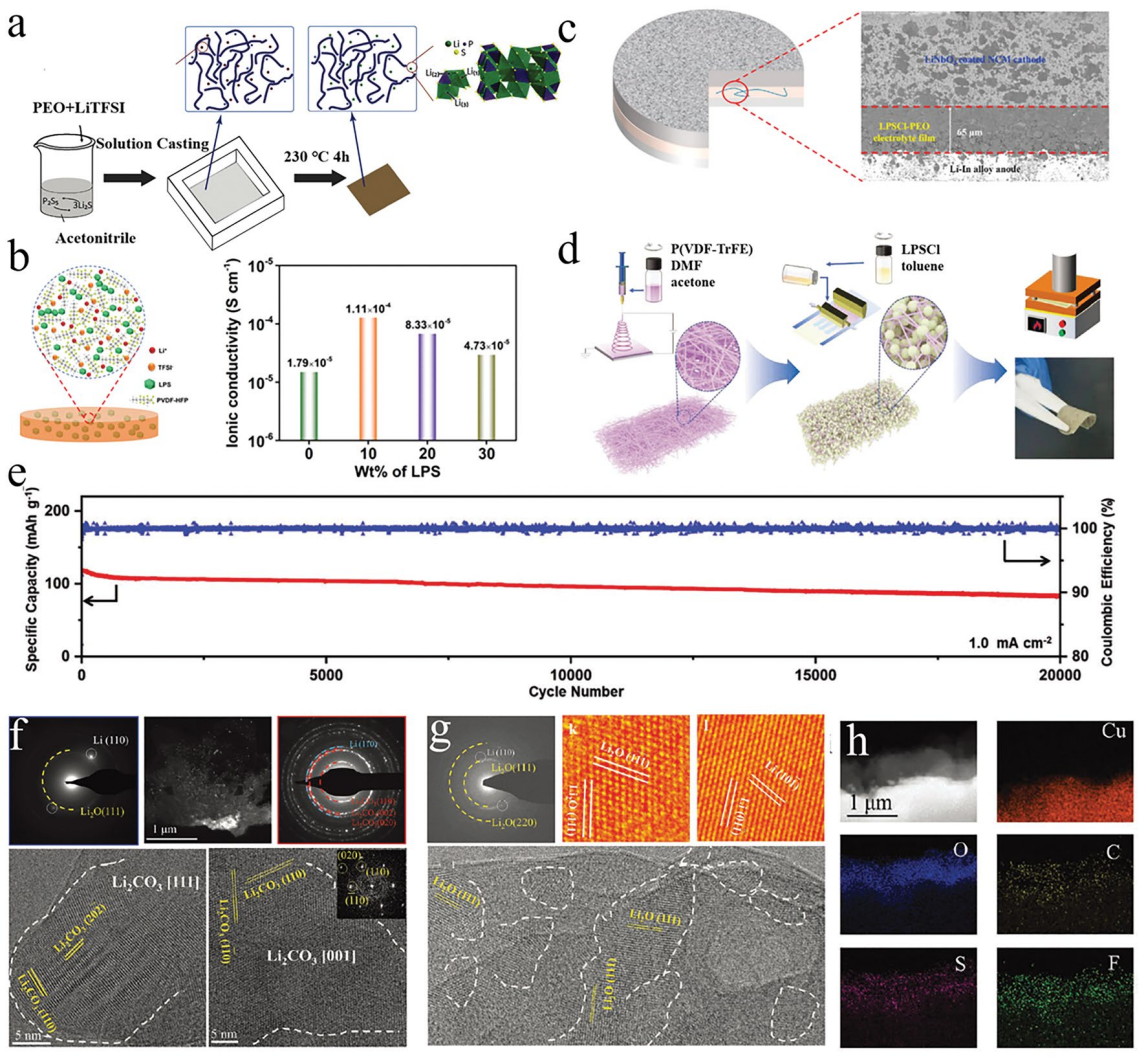
**a** Process flow diagram of in-situ preparation of PEO-Li_3_PS_4_ OICSE [188], Copyright 2018 Elsevier. **b** Schematic illustration of OICSE and Arrhenius plots of Li_7_PS_6_, OICSE, and PVDF-HFP/LiTFSI polymer electrolyte. [189], Copyright 2020, American Chemical Society. **c** Cycling performance of modified Li_6_PS_5_Cl-PEO and Li in alloy cathodes [190], Copyright 2020 Elsevier. **d** Schematic illustration of LPSCl@P(VDF-TrFE) OICSEs via an electrospinning-infiltration hot-pressing method. **e** Long-term cycling performance of LPSCl@P(VDF-TrFE) OICSEs at 1.0 mA cm^−2^ [191], Copyright 2022 Wiley. **f** Cryo-TEM characterization of the Li/PEO interfaces. **g** Cryo-TEM characterization of the Li/S-CPE interfaces. **h** EDS elemental maps of S-CPE [192], Copyright 2022 Wiley

Xu et al. developed an OICSE by incorporating Li_10_GeP_2_S_12_ (LGPS) as an active filler into a PEO matrix. The OICSE with 1 wt% LGPS exhibited higher ionic conductivity than that of the PEO-LiTFSI electrolyte, with values of 1.18 × 10^−5^ S cm^−1^ at 25 °C and 1.21 × 10^−3^ S cm^−1^ at 80 °C, and had a wide electrochemical window of 5.7 V versus Li^+^/Li [187]. This result is attributed to the inhibition of PEO crystallization by LGPS, which weakens the interaction between Li^+^ and PEO chains. Furthermore, adding LGPS particles to the PEO matrix enhanced the Li^+^ transference number and electrochemical stability. The LFP||Li batteries using the PEO-LiTFSI-1 wt% LGPS OICSE demonstrated a high capacity of 148.6 mAh g^−1^ at 0.5 C and 60 °C, with a capacity retention of 92.5% after 50 cycles. To further improve the uniform dispersion of nanofillers within a polymer matrix, Xu et al. introduced a novel in-situ synthesis method for Li_3_PS_4_ to create a PEO/Li_3_PS_4_ OICSE, as shown in Fig. 8a [188]. The results show that the in-situ synthesized Li_3_PS_4_ nanoparticles exhibit superior dispersion within the PEO matrix than mechanical mixing, which is conducive to forming Li^+^ conductive channels and enhancing ion transport. Specifically, the OICSE containing 2 vol% Li_3_PS_4_ by in-situ synthesized method demonstrated the highest ionic conductivity of 8.01 × 10^−4^ S cm^−1^ at 60 °C, surpassing the ionic conductivity of mechanically mixed electrolytes at 6.98 × 10^−4^ S cm^−1^. Additionally, the assembled solid-state LiFePO_4_/Li battery with the OICSE displayed outstanding cycling performance with a capacity retention of 80.9% after 325 cycles at 60 °C and remarkable rate capability (127 mAh g^−1^ at 1 C). In efforts to enhance the chemical stability of sulfides in an air environment, as well as to improve electrode material compatibility, Wang et al. have successfully designed a novel sulfide-doped OICSE. This OICSE combines inorganic sulfide, specifically lithium-sulfur saltpeter (Li_7_PS_6_), with a polyvinylidene fluoride-hexafluoropropylene copolymer (PVDF-HFP) [189]. Incorporating Li_7_PS_6_ within a PVDF-HFP polymer matrix imparts flexibility and air stability to the OICSE while ensuring commendable chemical and electrochemical stability. Notably, the PVDF-HFP-Li_7_PS_6_ electrolyte exhibited excellent ionic conductivity of 1.1 × 10^−4^ S cm^−1^ at RT (Fig. 8b), and the Li||Li symmetric cell achieved stable cycling of up to 1000 h at 0.2 mA cm^−2^. In addition, the LiFePO_4_||CSE||Li cell displays an impressive specific capacity of 160 mAh g^−1^ over 150 cycles, indicating that sulfide-doped OICSEs are promising for high-performance solid-state lithium batteries. Zhang et al. engineered a thin sulfide electrolyte film (65 μm) through the modified Li_6P_S_5_Cl and PEO, as shown in Fig. 8c [190]. The assembled Li-In ||LiNi_0.7_Co_0.2_Mn_0.1_O_2_ ASSLBs with the OICSE exhibit 74% capacity retention and an average coulombic efficiency of 99.85% after 1000 cycles at 60 °C with high loading conditions (4.46 mAh cm^−2^). Liu et al. prepared ultrathin flexible OICSE from Li_6_PS_5_Cl and poly(vinylidene fluoride-trifluoroethylene) (P(VDF-TrFE)) through the electrostatic spinning infiltration-hot-pressing method, shown in Fig. 8d [191]. The strong polarity of the polymer facilitates the interaction with LSPSCl. The P(VDF-TrFE) network allows full penetration of the LPSCl particles and the formation of an interpenetrating P(VDF-TrFE) structure. The ionic conductivity reached 1.2 × 10^−3^ S cm^−1^, enabling the Li-In||LiNi_0.8_Co_0.1_Mn_0.1_O_2_ cell to maintain 71% capacity after 20,000 cycles at 1.0 mA cm^−2^ (Fig. 8e). To inhibit the growth of polysulfide shuttles and lithium dendrites, Su et al. designed an ASSLB with a flexible composite cathode and PEO-LSPSCl-LiTFSI (S-CPE) [192]. The cell still maintained 97.8% capacity retention after 100 cycles of 0.1 A g^−1^. Low-temperature transmission electron microscopy (Cryo-TEM) revealed the presence of abundant Li_2_CO_3_ particles at the Li/PEO interface (Fig. 8f), which hindered the Li^+^ transport. However, at the Li/S-CPE interface, LSPSCl promoted the decomposition of TFSI^−^ to form abundant Li_2_O nanocrystals, amorphous LiF, and Li_2_S layers, which suppressed the Li dendrites growth of and stabilized the interface (Fig. 8g). Furthermore, the comprehensive elemental mapping through EDS unveiled the distinct presence of elemental constituents such as O, F, S, and C within the structure of S-CPE (Fig. 8h). It is notably imperative to highlight that the pronounced O signal strength in the analysis suggests an intricate process involving Li deposition coupled with the formation of Li_2_O. This work provides a strategy to mitigate the polysulfide shuttle effect and lithium dendrite formation for the design of solid-state lithium-based batteries with high energy density. Table 5 summarizes typical examples of the electrochemical performance of OICSE with sulfide-type fillers.

**Table 5:** Electrochemical properties of OICSEs with sulfide-type materials

Fillers	OICSEs	δ (S cm^−1^)	T_*Li*+_	EW (V)	References
Li_10_GeP_2_S_12_	PEO/1% LGPS	1.18 × 10^−5^ (25 °C)	0.26	5.7	[187]
Li_3_PS_4_	PEO-2 vol% Li_3_PS_4_	8.01 × 10^−4^ (60 °C)	0.33	5.1	[188]
Li_6.25_PS_5.25_Cl_0.75_	1 wt% LiPSCl /PEO	1.2 × 10^−2^ (25 °C)		5.1	[193]
β-Li_3_PS_4_	PGMA-50% LPS	1.8 × 10^−4^ (RT)		4.8	[194]
Li_10_SnP_2_S_12_	PEO-1% Li_10_SnP_2_S_12_	1.69 × 10^−4^ (50 °C)	0.38	5	[195]
LPS	LPS -LiTFSI- PVDF	3.42 × 10^−4^ (RT)	0.44		[196]
Li_3.25_Ge_0.25_P_0.75_S_4_	LGPS /PEO	0.42 × 10^−3^ (20 °C)	0.87		[197]
Li_7_PS_6_	Li_7_PS_6_- PVDF-HFP	1.1 × 10^−4^ (RT)			[189]
Li_6_PS_5_Cl	PEO_20_: LiTFSI-40% Li_6_PS_5_Cl	3.6 × 10^−3^ (80 °C)			[198]
78Li_2_S-22P_2_S_5_	78Li_2_S-22P_2_S_5_- PVDF -LiTFSI	4.7 × 10^−4^ (RT)			[199]
Li_6_PS_5_Cl	Li_6_PS_5_Cl-5% PEO-LiClO_4_	3.0 × 10^−3^ (25 °C)		4.2	[190]
Li_6_PS_5_Cl	LPSCl-PEO_3_-LiTFSI	1.1 × 10^− 3^ (25 °C)		4.9	[200]
Li_10_GeP_2_S_12_	LGPS-PVDF	2.64 × 10^−4^ (50 °C)		5.0	[201]
Li_10_GeP_2_S_12_	LGPS -PEO- LiTFSI	2.4 × 10^−4^ (25 °C)		4.7	[202]
Li_6_PS_5_Cl	P(VDF-TrFE)- Li_6_PS_5_Cl	1.2 × 10^−3^ (RT)		5	[191]
Li_10_Si_0.3_PS_6.7_Cl_1.8_	PEO-LSPSCl- LiTFSI	4.7 × 10^−3^ (70 °C)			[192]
LPSCl	12 wt% PPO-LPSCl	3.0 × 10^−4^ (RT)			[203]

#### Polymer Matrix Incorporating Garnet-Type Materials

The garnet-type solid-state electrolyte materials are typically lithium-ion conductors like Li_7_La_3_Zr_2_O_12_ (LLZO) and their derivatives. They are known as fast ion conductors, exhibiting relatively high ionic conductivity from 10^−4^ to 10^−3^ S cm^−1^ [204]. When using LLZO particles, it is important to ensure that the surface is fresh, as Li_2_CO_3_ and LiOH are easily formed when exposed to the air. In addition, these materials possess a wide electrochemical window, outstanding chemical stability, excellent mechanical strength, and the ability to suppress lithium dendrite growth effectively. Incorporating garnet-type fillers within polymer electrolytes has demonstrated promise in mitigating the issues associated with lithium dendrite growth while enhancing the overall electrochemical performance.

**Fig. 9:**
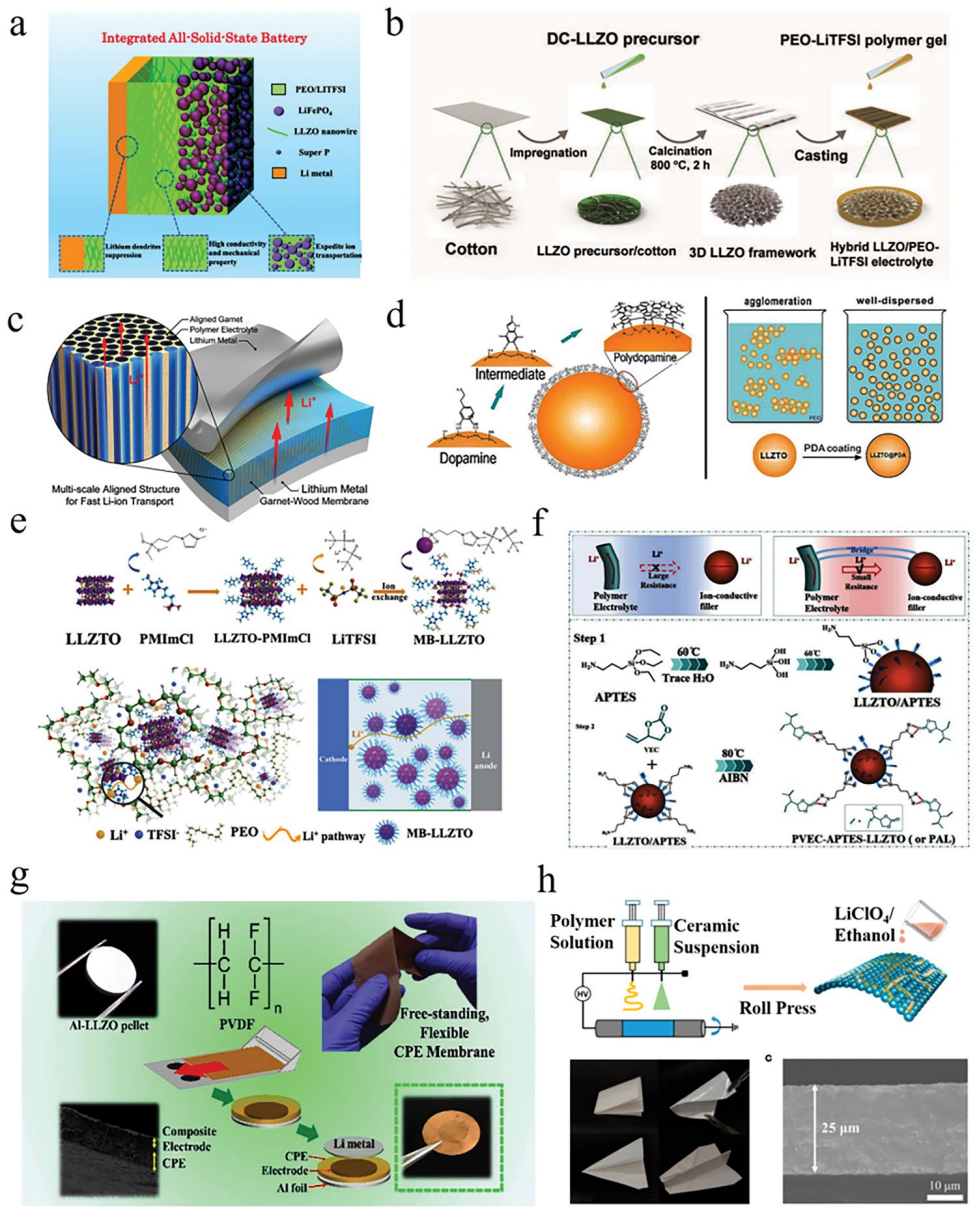
**a** Schematic illustration of an integrated LiFPO_4_/CSE/Li battery [206], Copyright 2018 WILEY. **b** Schematic illustration for the preparation of LLZO/PEO-LiTFSI electrolyte [207] Copyright 2019 WILEY. **c** Schematic of multiscale aligned mesoporous garnet LLZO membrane incorporated with PEO polymer [208], Copyright 2019, American Chemical Society. **d** Schematic diagram of dopamine polymerization on the LLZTO surface to form a polydopamine coating and the dispersion of LLZTO particles (coated and uncoated with PDA) in PEO solution [209], Copyright 2019 Royal Society of Chemistry. **e** Schematic diagram of the synthesis route for grafting molecular brushes onto LLZTO surface (MB-LLZTO) [211], Copyright 2019 Royal Society of Chemistry. **f** Preparation process diagram of an OICSE that forms a "bridge" between polymer and ceramic phase [212], Copyright 2023 Elsevier. **g** Schematic diagram of tape casting and battery manufacturing of PVDF/Al LLZO film on composite electrodes [214], Copyright 2023, American Chemical Society. **h** Preparation method and characterization diagram of PAN/LiClO_4_: LLZTO film [215], Copyright 2020, American Chemical Society

Lee et al. [205] evaluated the ionic conductivity of OICSEs, consisting of different tetragonal LLZO contents with PEO matrix. The results showed that the OICSE containing 52.5 wt% LLZO indicated the highest ionic conductivity of 4.42 × 10^−4^ S cm^−1^ at 55 °C and was higher than that of the OICSE containing 52.5 wt% Al_2_O_3_ inert material (10^−6^ S cm^−1^). This phenomenon arises from the synergistic effect resulting from the combination of the polymer and the active LLZO filler, consequently enhancing the ionic conductivity. Goodenough et al. compared the ionic conductivity of OICSEs prepared from SiO_2_ and LLTO nanoparticles as inert and active fillers, respectively [69]. The OICSE with LLTO nanoparticle showed an ionic conductivity of 1.9 × 10^−5^ S cm^−1^, which is twice times higher than OICSE with SiO_2_ nanoparticle. This improvement of LLTO nanoparticles is due to the fast interphase conduction between the active filler and the PEO matrix. Most importantly, the OICSE with LLTO framework showed the highest ionic conductivity of 8.8 × 10^−5^ S cm^−1^ at 25 °C, which was higher than that of both active (LLTO particles) and inert (SiO_2_ particles) fillers. This is due to the 3D framework with PEO can provide continuous ion transport channels compared to nanoparticles, avoiding the accumulation of particles, and thus effectively improving the ion transport properties. He et al. achieved high ionic conductivity of 2.39 × 10^−4^ S cm^−1^ at 25 °C by incorporating LLZO nanowires into a PEO electrolyte (PLLN), and a wide electrochemical window of 6 V versus Li^+^/Li was measured by LSV using Li|OICSE|SS, as shown in Fig. 9a [206]. The tensile strength of PLLN increases to nearly 1.0 MPa with a maximum strain of 2092% owing to the high rigidness and good dispersity of LLZO nanowires. The all-solid-state LFP/PLLN/Li batteries exhibit a favorable specific capacity of 158.8 mAh g^−1^ after 70 cycles at 0.5 C under 60 °C and a specific capacity of 158.7 mAh g^−1^ after 80 cycles at 0.1 C under 45 °C. The uniform dispersion of LLZO nanowires in the polymer led to a significant enhancement in both the ionic conductivity and mechanical strength of the OICSE. The Li||Li symmetric battery assembled by the OICSE exhibits stable cycling performance for 1000 h at 60 °C without a short circuit. Li et al. synthesized 3D garnet-type LLZO monomers by employing skimmed cotton as a template for fabricating flexible solid-state LLZO/PEO LiTFSI electrolytes (Fig. 9b) [207]. This OICSE achieves ionic conductivity of 0.89 × 10^−4^ S cm^−1^ and exhibits a wide electrochemical window of 5.5 V versus Li^+^/Li using Li|OICSE|SS. ASSLBs matched with LiFePO_4_ exhibited high cycle stability and rate performance. To reduce the tortuosity of the ion conduction path, Hu et al. employed wood as a template, in conjunction with the polymer PEO, to fabricate a garnet framework structure with a highly conductive multiscale arrangement from a top-down approach (Fig. 9c) [208]. The structure exhibits an impressive ionic conductivity of 1.8 × 10^−4^ S cm^−1^ and excellent mechanical flexibility at RT. Notably, the ionic conductivity closely approximates the bulk conductivity, and the impact of the garnet/polymer interface is significantly amplified. The low-curvature garnet wood structure, serving as a highly conductive solid-state electrolyte, demonstrates substantial potential and offers a valuable model for research aimed at the design and optimization of OICSEs.

Improving the interfacial compatibility between nanofillers and polymers through surface modification is an effective method to enhance thermal stability and electrochemical properties. Huang and co-workers reported the modification of LLZTO nanoparticles by coating with polydopamine (PDA) [209]. Due to the dual wettability properties of dopamine on organic and inorganic materials, a strong bond was formed between LLZTO and PEO, as shown in Fig. 9d resulting in 80 wt% LLZTO uniformly dispersed in a polymer electrolyte composed of 20 wt% PEO/LiTFSI. The ionic conductivity increased from 6.3 × 10^−5^ S cm^−1^ to 1.1 × 10^−4^ S cm^−1^ at 30 °C after modification compared to unmodified LLZTO in PEO. Previous studies have indicated that 10–20 wt% LLZTO is well dispersed in PEO-based polymer electrolytes when LLZTO is unmodified [32, 64, 210]. Above this percolation value, particles begin to agglomerate, resulting in a decrease in ionic conductivity. Therefore, surface modification can improve the dispersibility of fillers by adding higher content fillers without agglomeration, thereby enhancing the mechanical strength and ion transport pathway of OICSEs. The surface modifying groups are usually acidic surface groups (e.g., -hydroxyl (–OH) groups), which enhance the interaction of the filler with the lithium salt and the polymer through hydrogen bonding, increasing the dissociation of the lithium salt. Or positively charged modifications (e.g., –NH_3_^+^ groups), which improve the anion adsorption capacity of the filler through electrostatic interactions and promote the dissociation of the lithium salt, thus increasing the ionic conductivity and Li^+^ transference number. Li et al. employed a molecular brush modification LLZTO approach, denoted as MB-LLZTO. They utilized 1-methyl-3-trimethoxysilane imidazolium chloride (PMImCl) and incorporated MB-LLZTO into the PEO matrix to create OICSE, as illustrated in Fig. 9e [211]. The results showed that the OICSE containing 15 wt% MB-LLZTO exhibited the highest ionic conductivity of 3.11 × 10^−4^ S cm^−1^ at 45 °C. This represented a significant improvement from the ionic conductivity of pristine LLZTO-CPE (9.16 × 10^−5^ S cm^−1^). The all-solid-state lithium-sulfur battery with MB-LLZTO-CPE shows the highest discharge capacity of 1280 mAh g^−1^ at low temperature and stable cycling performance (752 mAh g^−1^ after 220 cycles). The construction of molecular brushes on the LLZTO surface may be an effective way to unlock more potential of solid polymer electrolytes. Yu et al. reduced the interfacial resistance and increased the electrochemical window by creating a "bridge" between the polymer and ceramic phases, as shown in Fig. 9f [212]. Chemical and hydrogen bonds between the polymer and ceramic phases were created, establishing ultrafast Li-ion transport channels. This structure resulted in high ionic conductivity of 3.1 × 10^−3^ S cm^−1^ at RT, and the symmetrical Li||Li cells exhibited long-life stripping/plating behavior over 1000 h at 0.1 mA cm^−2^ without short-circuiting. The LFP|PAL|Li battery shows a stable discharge capacity of 143 mAh g^−1^ and keeps 92% capacity retention over 100 cycles with a coulombic efficiency of 99% at 0.5 C. This “bridging” strategy provides an effective way to solve high interface resistance and interface compatibility problems.

In addition to PEO, polymers like PEGDA, PVDF, PAN, and PMMA are also incorporated into ceramic fillers to create various OICSEs. Yu et al. developed PEGDA-SN-LiTFSI-LLZTO electrolytes by incorporating LLZTO nanoparticles into a PEGDA polymer matrix [213]. The OICSE achieves a high ionic conductivity of 3.1 × 10^−4^ S cm^−1^ at RT, coupled with a wide electrochemical stability window of 4.7 V versus Li^+^/Li using Li|OICSE|SS. The LLZTO enhances ionic conductivity and helps suppress lithium dendrite formation. Concurrently, the PEGDA polymer ensures robust interfacial contact with the electrode. A flexible ceramic-polymer electrolyte composed of aluminum-doped garnet (Li_6.28_Al_0.24_La_3_Zr_2_O_12_) and PVDF at an 8:2 ratio, as shown in Fig. 9g [214]. This OICSE membrane indicates a broad electrochemical window of 5.5 V versus Li^+^/Li by LSV using Li|OICSE|SS, high ionic conductivity of 5 × 10^−5^ S cm^−1^, and Li^+^ transference number (0.69), outstanding mechanical strength, and thermal stability. The LFP |CPE|Li cell delivered an initial capacity of 137 mAh g^−1^ at 0.2 C and 121 mAh g^−1^ at 1 C with minimum resistance. Chen et al. prepared ultrathin PAN/LiClO_4_: LLZTO electrolytes using a combined electrospinning/electrospraying technique, which resulted in continuous interfacial conduction channels, as illustrated in Fig. 9h [215]. The OICSE exhibited a high ionic conductivity of 1.16 × 10^−3^ S cm^−1^ at 25 °C. Li-symmetric batteries employing this electrolyte achieved stable operation for up to 5000 h at very low overpotentials and without short-circuiting. The Li-CNT| OICSE |LNMO cell exhibits a specific capacity of 137.2 mAh g^−1^ at a current of 0.25 C with a capacity retention of 93.0% after 180 cycles. This indicates that the OICSE reported in this work can sustain stable cycling with high voltage LNMO for high energy density lithium metal batteries. Understanding the Li^+^ ion transport mechanism in polymer ceramic systems can provide new insights into the structural design of OICSEs. The ion transport mechanism section of Chapter 3 has already been described in detail. Therefore, it will not be repeated here. Additionally, Table 6 summarizes the electrochemical properties of OICSEs with garnet-type materials.

**Table 6:** Electrochemical properties of OICSEs with garnet-type materials

Fillers	OICSEs	δ(S cm^−1^)	T_*Li*+_	EW(V)	References
LLZO	PEO/52.5 wt% LLZO	4.42 × 10^−4^ (55 °C)		5	[205]
LLZTO	PEO/LLZAO/LiTFSI	2.5 × 10^−4^ (RT)		6	[65]
LLZTO	PEO: LLZTO	2.1 × 10^−4^ (30 °C)		4.7	[61]
LLZO	PEO-LiTFSI 7.5 wt% LLZO	5.5 × 10^−4^ (30 °C)	0.207	5.7	[216]
LLZTO	PEO/LLZTO/LiTFSI	5.2 × 10^−4^ (20 °C)	0.75	4.6	[87]
LLZTO	10 wt% LLZTO/PVDF	5 × 10^−4^ (25 °C)			[53]
Al-doped LLZTO	PEO(LiTFSI)- 40 wt% LLZTO	1.12 × 10^−5^ (25 °C)	0.58	5.5	[81]
Ta-doped LLZO	PAN-LiClO_4_-5 wt% LLZO	1.31 × 10^−4^ (RT)	0.3		[92]
LLZTO	PEO- LLZTO -PEG-60 wt% LiTFSI	6.24 × 10^−5^ (25 °C)		5	[32]
LLZTO	LiTFSI/PVDF/LLBZTO/LiF	3.4 × 10^−4^ (20 °C)			[100]
LLZAO	PEO-aligned LLZAO	1.8 × 10^−4^ (RT)			[208]
LLZO	PEO- 40 wt% LLZO	1.0 × 10^−4^ (20 °C)	0.5	4.9	[217]
LLZTO	LLZTO@PDA/PEO	1.15 × 10^−4^ (30 °C)		4.8	[209]
LLZTO	PISE-10% LLZTO	2.13 × 10^−4^ (25 °C)	0.57	4.9	[218]
LLZTO	PAN/LiClO_4_: LLZTO	1.16 × 10^−3^ (25 °C)		4.9	[215]
LLZTO	LLZTO/PVDF	1.23 × 10^−4^ (25 °C)	0.51	4.8	[219]
LLZTO	PEGDA-SCN-LiTFSI-LLZTO	3.1 × 10^−4^(RT)	0.43	4.7	[213]
LLZO	PVDF-PEO-LiTFSI-LLZO	1.05 × 10^−4^(50 °C)	0.45	5.0	[220]
LLZO	PEO-10 wt% pLLZO	1.36 × 10^−5^(25 °C)			[57]
LLZTO	PEO-SN-LLZTO	3.5 × 10^−4^ (30 °C)	0.65	5.5	[221]
LLZO	PPO-45 wt% LLZO	3.75 × 10^−4^ (25 °C)	0.67	5	[222]
LALZ	PVDF-5 wt% LALZ	5 × 10^−5^ (RT)	0.69	5.5	[214]
Al-doped LLZO	PVDF-HFP-PPC-LLZO	4.04 × 10^−4^ (RT)	0.58	4.9	[223]
LLZTO	PVEC-LLZTO	3.1 × 10^−3^ (25 °C)	0.64		[212]
LLZTO	PEO-SN-LLZTO	6.74 × 10^−4^ (RT)		4.7	[88]
LLZO	PEO-10 wt% LLZO nanowires	2.39 × 10^−4^ (RT)			[206]
LLZTO	PEO-10 wt% LLZTO				[224]

#### Polymer Matrix Incorporating NASICON-Type Materials

The general structural formula for NASICON-type fast ion conductors is AM_2_(PO_4_)_3_, where 'A' denotes a monovalent metal cation (e.g., Li^+^, Na^+^, K^+^) and 'M' signifies a tetravalent or trivalent metal cation (such as Ge^4+^, Al^3+^, Ti^4+^). Among these materials, Li_1.3_Al_0.3_Ti_1.7_(PO_4_)_3_ (LATP) and Li_1.5_Al_0.5_Ge_1.5_(PO_4_)_3_ (LAGP) are particularly noteworthy, having garnered extensive research interest for their exceptional ionic conductivity at RT (on the order of 10^−3^ S cm^−1^) and a broad electrochemical stability window (~ 5 V). Nevertheless, the reactivity of Ti^4+^ and Ge^4+^ with lithium metal may elevate interfacial impedance, compromising stability. Integrating polymer electrolytes has been recognized as a viable strategy to bolster interface stability and enhance the overall electrochemical performance of NASICON-type electrolytes.

**Fig. 10:**
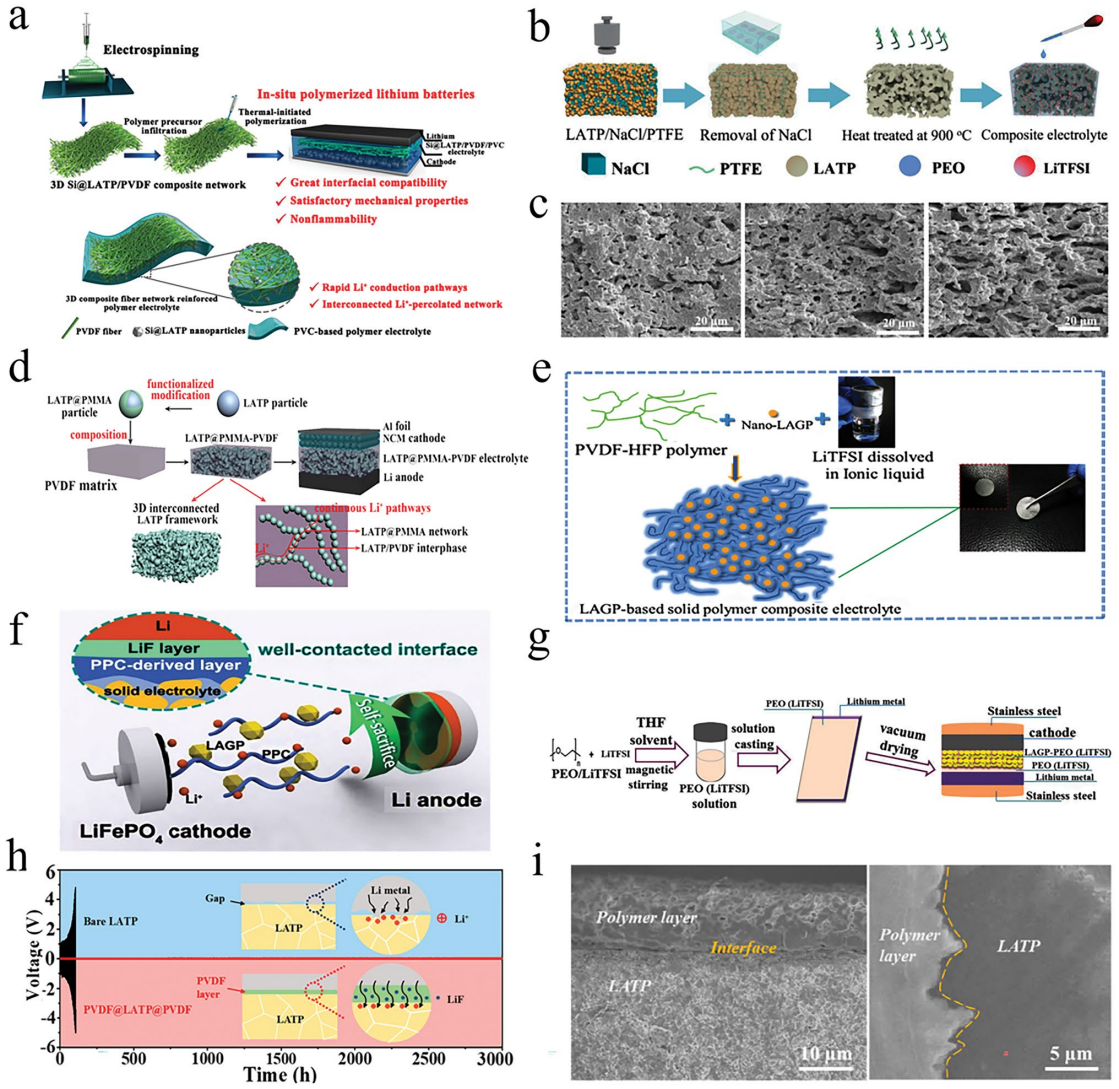
**a** Schematic diagram of 3D composite fiber network reinforced CPE preparation [226], Copyright 2022 Elsevier. **b** Schematic diagram of 3D porous LATP framework. **c** SEM images of 3D porous LATP frameworks with different NaCl template mass fractions [227], Copyright 2021 Elsevier. **d** Schematic diagram of the synthesis process and conduction mechanism of LATP@PMMA-PVDF electrolytes [228], Copyright 2021 Elsevier. **e** Schematic illustration for the synthesis of OICSE [229], Copyright 2017, American Chemical Society. **f** Schematic diagram of LiFePO_4_ | LAGP/30% PPC | Li batteries forming a LiF protective layer [230], Copyright 2019, American Chemical Society. **g** Schematic diagram of Li/PEO (LiTFSI)@LAGP-PEO (LiTFSI)/LiMFP batteries preparation [231], Copyright 2017, American Chemical Society. **h** Schematic diagram of LATP and PVDF@LATP@PVDF electrolytes at 0.1 mA cm^−2^. **i** Cross-sectional and surface SEM images of the LATP pellet [232], Copyright 2022, American Chemical Society

Wang et al. synthesized LATP-PEO hybrid electrolytes utilizing a solution casting technique. Employing electrochemical impedance spectroscopy (EIS), the optimum ionic conductivity for LATP-PEO with an EO/Li ratio of 16 was 2.631 × 10^−6^ S cm^−1^ at RT [225]. By augmenting the system with lithium salts, the room-temperature ionic conductivity of PEO- LiClO_4_-LAGP, with an EO/Li ratio of 8 and containing 15 wt% LAGP, was enhanced to 7.985 × 10^−6^ S cm^−1^. Xing et al. developed a 3D silane-modified LATP /PVDF composite electrolyte with Li^+^-percolated conductive networks through electrostatic spinning (Fig. 10a) [226]. The 3D Si@LATP/PVDF OICSE demonstrated superior ionic conductivity of 1.06 mS cm^−1^ at 25 °C, a large Li^+^ transference number of 0.82. The significant enhancement of the Li^+^ transference number is attributed to the positive charge of -NH_3_^+^ in the polysiloxane grafted onto the LATP, which makes the surface of Si@LATP positively charged and fully exposes the Lewis acid sites of the LATP, thus enhancing the anion adsorption capacity of the LATP based on electrostatic interactions. Moreover, the nanofibrous architecture significantly enhances the strength of polymer matrix, the 3D Si@LATP/PVDF OICSE has a high tensile strength of 15.3 MPa and a wide electrochemical window of 4.86 V versus Li^+^/Li was measured through LSV using Li|OICSE|SS cell. Nevertheless, the current synthesis requires specialized equipment, posing certain limitations and prompting interest in simpler, equally effective production methods for three-dimensional, high-strength skeletal electrolytes. Fan et al. successfully developed a new and simple 3D LATP porous conductive framework using common and inexpensive NaCl powder as a sacrificial template (Fig. 10b) [227]. This approach is not only facile and low-cost but also environmentally friendly because the template can be dissolved in water, and the porosity of the 3D porous conductive framework is easily controlled (Fig. 10c). Integrating a PEO matrix resulted in a 3D LATP-PEO electrolyte with notable ionic conductivity of 7.47 × 10^−4^ S cm^−1^ at 60 °C. The symmetrical Li||Li cells with this electrolyte exhibited long-life stripping/plating behavior over 1000 h at 0.2 mA cm^−2^. To avoid the accumulation of particles and to improve the filler–polymer interaction, Xiong et al*.* engineered PMMA-coated LATP with PVDF matrix, as shown in Fig. 10d [228]. The molecular affinity between PMMA and PVDF facilitated a uniform dispersion of PMMA-coated LATP particles throughout the polymer matrix, resulting in a continuous and interconnected 3D LATP network. In addition, the enhanced affinity of LATP for the PVDF matrix and the inherent Li^+^ complexation ability of PMMA ensured straight Li^+^ conduction channels through the LATP framework and the LATP/PVDF interface. The results showed that the LATP@PMMA-PVDF electrolyte obtained a high ionic conductivity of 1.23 × 10^−3^ S cm^−1^ and a Li^+^ transference number of 0.85 at RT. Additionally, by incorporating ionic liquid salts into the LAGP/PVDF-HFP electrolyte, as illustrated in Fig. 10e, the interfacial wettability between the solid electrolyte and active materials was significantly improved, effectively enhancing the ionic conductivity of the OICSE [229]. This compatibility with lithium metal enabled LiFePO_4_ solid-state lithium batteries achieves discharge capacity as high as 157.8 mAh g^−1^ at 0.05 C and maintains 141.3 mAh g^−1^ after the 50th cycle with a capacity retention of 89.5%, offering a strategic and innovative approach to advancing solid-state battery technology.

To address the challenges associated with the high interfacial impedance and failure of solid-state batteries stemming from the incompatibility between NASICON-type inorganic electrolytes and lithium metal, the strategic formation of a spontaneous protective layer or the deliberate incorporation of a synthetic polymer layer presents a productive approach. Yu et al*.* designed a "self-sacrificing" interface through the reaction of a LiF layer deposited on a flexible LAGP/30% polypropylene carbonate (PPC) OICSE with Li metal, as shown in Fig. 10f [230]. This layer reduces the interfacial resistance between the electrolyte and the Li metal and endows the LiFePO_4_/Li cell with a discharge-specific capacity of 151 mAh g^−1^ at 0.05 C and a retention of 92.3% for 100 cycles at 55 °C. Wang et al*.* introduced a PEO (LiTFSI) interlayer between the LAGP-PEO CSE and lithium metal (Fig. 10g), mitigating side reactions at the interface [231]. The assembled Li-PEO (LiTFSI)/LAGP-PEO/LiMn_0.8_Fe0_.2_PO_4_ all-solid-state battery displayed an initial discharge capacity of 160.8 mAh g^−1^, with notable cycling stability and rate performance at 50 °C. Similarly, Tao et al. further advanced the field by implementing a PVDF buffer layer on the LAGP surface, successfully reducing the interfacial impedance (from 5789 to 271 Ω) and effectively preventing the side reactions between the Li anode and LATP [232]. The Li||Li symmetric battery exhibited an exceptionally long life of over 3000 h at 0.1 mA cm^−2^ (Fig. 10h**)**. Additionally, ASSLBs matched with LiFePO_4_ indicated robust cycling stability with an initial discharge capacity of 141.1 mAh g^−1^ and maintained 83.4% capacity after 300 cycles. SEM images in Fig. 10i confirm the uniform PVDF coating on LATP particles, suggesting favorable interfacial chemistry. The above results indicate that the interfacial modification strategy successfully protected the electrolyte and lithium metal instability. Meanwhile, it provides a helpful contribution to developing stable solid-state batteries.

#### Polymer Matrix Incorporating Perovskite-Type Materials

The fastest lithium-ion-conducting electrolytes are the perovskite-type La_2/3-x_Li_3x_TiO_3_ (LLTO) and its variants. These materials are characterized by their significant A-site vacancy concentrations, facilitating efficient Li-ion migration. They exhibit a bulk conductivity of 10^−3^ S cm^−1^ at ambient temperature, with grain-boundary conductivity ranging from 10^−4^ to 10^−5^ S cm^−1^, comparable to traditional liquid electrolytes. Nevertheless, these perovskite-type electrolytes demonstrate a propensity for reduced cathodic stability, a drawback primarily ascribed to their reactive reduction with lithium metal (Ti^4+^ + Li → Ti^3+^ + Li^+^).

**Fig. 11:**
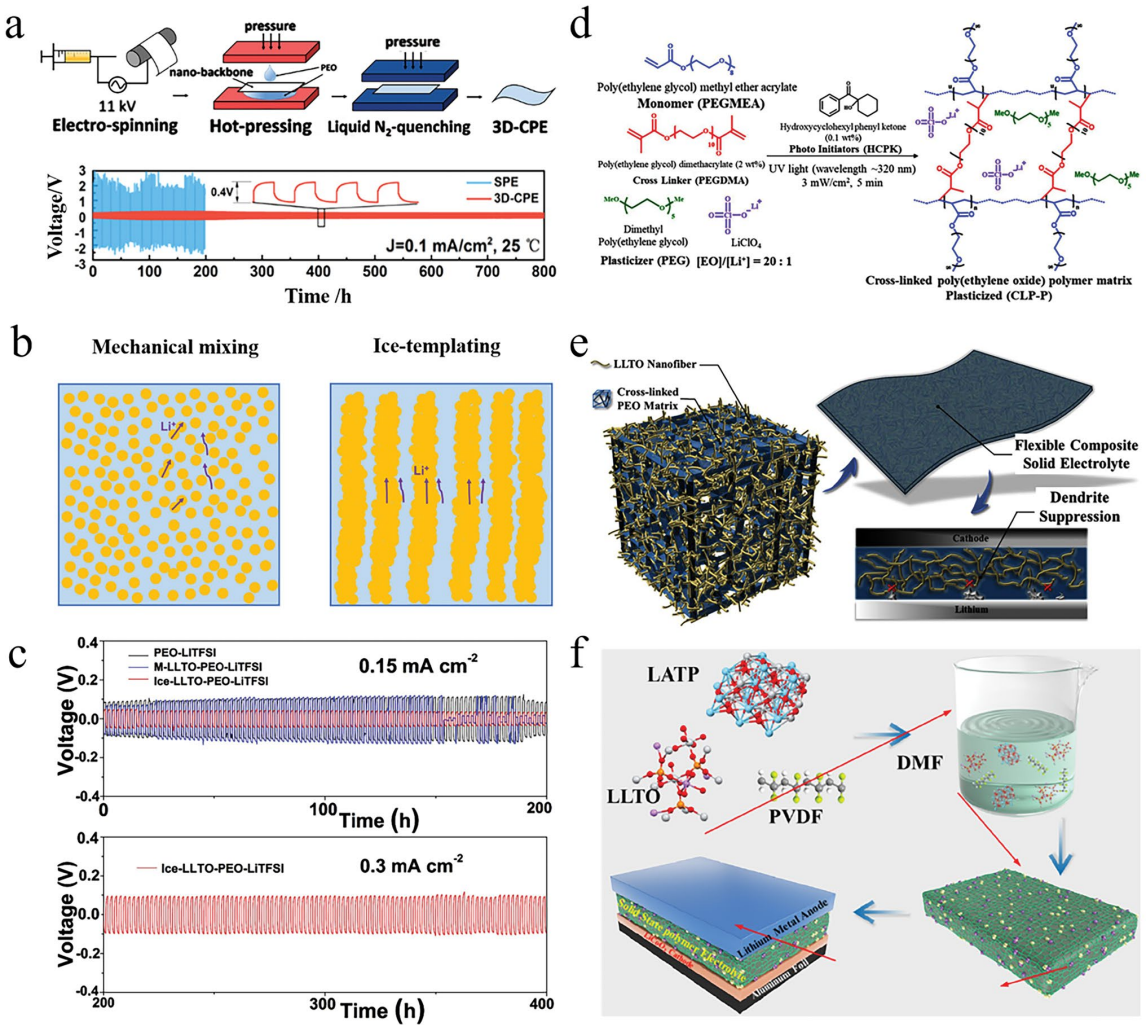
**a** Schematic diagram of 3D-CPEs preparation and Li plating and stripping cycling voltage profiles for the SPE and 3D-CPE [234], Copyright 2018, American Chemical Society. **b** Schematic diagram of ion transport paths for OICSE with mechanically mixed LLTO and OICSE with vertically aligned LLTO framework. **c** Li plating and stripping cycling voltage profiles for the PEO-LiTFSI and Ice-LLTO-PEO-LiTFSI [235], Copyright 2020 Elsevier. **d** Schematic diagram of cross-linked polyethylene oxide solid polymer electrolyte preparation. **e** Schematic diagram of three-dimensional fiber network OICSE composed of nanofibers and cross-linked polyethylene oxide solid polymer [236], Copyright 2019, Donghua University. **f** Schematic diagram of dual semi-solid polymer electrolyte films preparation [238], Copyright 2021, American Chemical Society

Analogous to polymer-garnet OICSEs, the ionic conductivity of polymer-perovskite OICSE is markedly influenced by particulate characteristics such as size, morphology, and spatial distribution. Table 7 summarizes the electrochemical properties of OICSEs with perovskite-type materials. Zhang *et al*. utilized the electrospinning technique to fabricate Li_0.33_La_0.557_TiO_3_ (LLTO) nanofibers with an elevated aspect ratio. Subsequently, they incorporated these into a PEO matrix to engineer a PEO/LLTO OICSE [233]. This innovative electrolyte demonstrated an ionic conductivity of 2.4×10^−4^ S cm^−1^ at RT and exhibited an impressive electrochemical window of 5 V versus Li^+^/Li in Li|OICSEs|SS cell. Nan et al. fabricated CPEs by incorporating a 3D LLTO nano-network into a PEO matrix through hot-pressing and quenching (Fig. 11a) [234]. The CPE featuring the 3D LLTO network (3D-CPE) displayed a higher ionic conductivity of 1.81×10^−4^ S cm^−1^ and a wide electrochemical window of 4.5 V versus Li/Li^+^ at RT than that of the PEO-based electrolyte. This enhancement in ion transport efficiency is ascribed to the uniform distribution of interfaces between the LLTO framework and the PEO matrix, which facilitates a rapid ion transport pathway and reduces the barrier for ion hopping. With the help of the LLTO nanofiber network, the 3D-CPE exhibits a tensile strength of 16.18 MPa, Young's modulus of 0.98 GPa, elongation of over 200%, and an apparent yield point, which is attributed to the good adhesion between the matrix and filler and the strong support of the inorganic LLTO backbone. The symmetrical Li|3D-CPE|Li battery exhibited a long cycle life of over 800 h at 0.1 mA cm^−2^ (Fig. 11a), indicating that the 3D-CPE film can effectively inhibit the Li dendrites growth and is a promising candidate electrolyte for flexible solid-state lithium-ion batteries. Zhao et al. examined the effects of randomly dispersed LLTO nanoparticles and vertically aligned LLTO on the enhancement of ionic conductivity in PEO/LiTFSI/LLTO electrolytes (Fig. 11b) [235]. The OICSE with Ice-LLTO-PEO-LiTFSI structure achieves a remarkable ionic conductivity of 1.3×10^−4^ S cm^−1^, 2.4 orders of magnitudes higher than the mechanically mixed counterpart. The pronounced enhancement is attributed to the vertically aligned structure, which provides a contiguous and expedited network for the transport of Li^+^ ions. Furthermore, the symmetric Li||Li cell utilizing Ice-LLTO-PEO-LiTFSI OICSE demonstrates stable operation over 400 h at 0.3 mA cm^−2^ (Fig. 11c), Li|OICSE|LFP full battery delivers a specific discharge capacity of 144.6 mAh g^−1^ at 1 C at 60 °C with a high-capacity retention of 96.0% after 100 cycles, further confirming the superior electrochemical properties.

**Table 7:** Electrochemical properties of OICSEs with perovskite-type materials

Fillers	OICSEs	δ (S cm^−1^)	T_*Li*+_	EW(V)	References
Li_0.33_La_0.557_TiO_3_	PAN/LiClO_4_/15 wt% LLTO	2.4 × 10^−4^ (RT)			[64]
Li_0.33_La_0.557_TiO_3_	PAN/3wt% LLTO/ LiClO_4_	6.05 × 10^−5^ (30 °C)	0.42		[67]
Li_0.33_La_0.557_TiO_3_	PEO/LiTFSI/15 wt % 1D LLTO	2.4 × 10^−4^ (RT)		5	[233]
Li_0.33_La_0.557_TiO_3_	PEO-LiTFSI/3D LLTO	1.8 × 10^−4^ (RT)	0.33	4.5	[234]
Li_0.33_La_0.557_TiO_3_	PEO/3DLLTO	8.8 × 10^−5^ (RT)			[69]
Li_0.33_La_0.557_TiO_3_	PEO/LiClO_4_/3 wt% LLTO	4.01 × 10^−4^ (60 °C)	0.15	5.1	[239]
Li_0.33_La_0.557_TiO_3_	PEO/LiTFSI/3D LLTO	1.6 × 10^−4^ (24 °C)	0.48	4.7	[240]
Li_3/8_Sr_7/16_Ta_3/4_Zr_1/4_O_3_	PEO/LSTZO	3.5 × 10^−4^ (45 °C)	0.43		[241]
Li_0.33_La_0.557_TiO_3_	CLP-P4-30 wt% LLTO	3.31 × 10^−4^ (RT)	0.51	5	[236]
Li_0.33_La_0.557_TiO_3_	PEO/PPC/LiTFSI/8%LLTO	3.4 × 10^−4^ (60 °C)	0.17	5.1	[242]
Li_0.33_La_0.557_TiO_3_	PEO/LiTFSI/aligned LLTO	1.3 × 10^−4^ (RT)	0.55	4.75	[235]
Li_0.33_La_0.557_TiO_3_	PVDF/LLTO-CPE	5.8 × 10^−4^ (RT)		5.2	[243]
Li_0.33_La_0.557_TiO_3_	PVDF-HFP/10 wt% LLTO	1.21 × 10^−4^ (25 °C)	0.41	4.7	[244]
Li_0.33_La_0.557_TiO_3_	PEO-20 wt% LLTO	8.7 × 10^−4^ (60 °C)	0.51		[245]
Li_0.33_La_0.557_TiO_3_	PVDF/LATP/LLTO		0.88	5.3	[238]
Li_0.33_La_0.557_TiO_3_	3D PAN/LLTO NF	9.87 × 10^−4^ (30 °C)	0.41	4.8	[246]
Li_0.33_La_0.557_TiO_3_	PVDF-HFP/LLTO		0.62	4.8	[237]

To improve the ionic conductivity of the OICSE and reduce the large electrode/electrolyte interface impedance, Yan et al. synthesized a novel PEO-based crosslinked polymer (CLP) as a polymer matrix [236]. Figure 11d shows the crosslinking synthesis process of the CLP polymer matrix, in which poly(ethylene oxide) methyl ether acrylate (PEGMEA) monomer and poly(ethylene oxide) dimethacrylate (PEGDMA) were used as the crosslinking agents for photoinitiated polymerization. A certain amount of PEG plasticizer was added to improve the ion migration rate. LLTO nanofibers were doped into CLP matrix to form CLP-P-LLTO electrolyte (Fig. 11e), and the total ionic conductivity of CLP-P-LLTO was enhanced from 2.40 × 10^−4^ to 3.31 × 10^−4^ S cm^−1^ at RT. The CLP-P-LLTO delivered a noteworthy specific capacity of 147 mAh g^−1^ in the Li|LiFePO_4_ battery, and no significant lithium dendrite formation was observed at the anode/electrolyte interface after 100 cycles. Chang et al. introduced a fluorine-rich intercalation (denoted as succinonitrile interlayer) based on butanedinitrile, ethylidene fluorocarbonate, and LiTFSI in an LLTO/PVDF-HFP/LiTFSI electrolyte, which successfully reduced the interface resistance and suppressed unfavorable interfacial side reactions [237]. On the Li metal electrode, the SNI-derived solid electrolyte interface (SEI) enriched with LiF and CF_x_ slowed the build-up of dead lithium and excess SEI. Notably, the introduction of SNI significantly reduced the hydrogen defluorination reaction of PVDF-HFP. Siyal et al. proposed an innovative strategy to address the critical issues of lithium dendritic growth and interfacial resistance in lithium metal batteries [238]. They developed a dual semi-solid-state polymer electrolyte (DSPE) membrane by incorporating NASICON-type LATP and perovskite-type LLTO nanoparticles as Li^+^ ion-conducting ceramic fillers within a PVDF matrix (Fig. 11f). The results showed that this DSPE membrane successfully reduced interfacial impedance and protected lithium dendrites in lithium metal batteries. The symmetrical cell Li|DSPE|Li exhibits excellent stability at a high current density of 1 mA cm^−2^ over 1000 h, and the LiCoO_2_|DSPE|Li cell reaches an initial discharge specific capacity of 145.3 mAh g^−1^ at 0.1 C with a stable coulombic efficiency of 98% after 100 cycles. This provides a new method for preparing high-performance ASSLBs.

Table 8 summarizes the ionic conductivity, advantages, and disadvantages of active fillers. In OICSEs, the active material not only promotes lithium-ion transport by inhibiting the crystallinity of the polymer matrix but also participates directly in lithium-ion conduction. Therefore, choosing the active material requires comprehensive consideration of the following factors: ionic conductivity, chemical stability, mechanical properties, and compatibility with electrode materials. Optimization of these factors can achieve the best performance and sustainability of OICSEs.

**Table 8:** Summarize the ionic conductivity and advantages and disadvantages of active fillers

Type	Example	δ, S cm^−1^	Advantage	Disadvantage
Sulfide	LGPS LPS	10^−3^ to 10^−2^	High ionic conductivity Good interfacial contact Low interfacial resistance	Poor chemical stability, susceptible to air and moisture Poor interfacial contact with lithium metal
Garnet	LLZTO LLZO	10^−5^ to 10^−3^	Wide electrochemical window High mechanical strength Good compatibility with Li metal	High preparation costs Poor chemical stability, susceptible to air and moisture
NASICON	LATP LAGP	10^−5^ to 10^−3^	Good chemical stability Good mechanical strength Relatively simple preparation process	Poor interfacial contact with lithium metal
Perovskite	LLTO	10^−6^ to 10^−3^	High mechanical strength Good structural stability Wide electrochemical window	Expensive large-scale production Poor stability at high temperatures Poor compatibility with Li metal

## Advanced Characterization Method for OICSEs

As all-solid-state lithium metal batteries and other sophisticated energy storage systems advance, there is a burgeoning need for comprehensive research into composite electrolytes. This necessitates more intricate and nuanced characterization methods to reveal their structural complexities, intrinsic properties, and interfacial interactions. Therefore, some advanced material characterization techniques have important background and application value in OICSE research.

### Solid-State NMR Spectroscopy and Magnetic Resonance Imaging

Solid-state NMR and MRI techniques are non-destructive, quantitative, and qualitative. Over the past decades, solid-state NMR techniques have been widely used to study the structure and chain segment motions of composite electrolytes, polymer electrolytes, and polymer gel electrolytes [247]. The combination of magic angle rotation and broadband decoupling techniques has enabled high-resolution solid-state NMR spectroscopy, allowing the study of ionic interactions at the molecular level as well as information about the spatial proximity of functional groups. MRI is a powerful method for visualizing materials by encoding nuclear spin positions through magnetic field gradients, and in-situ MRI studies have contributed to the understanding of fundamental phenomena related to cell performance and failure mechanisms. Then, the application of solid-state NMR and MRI to the study of composite electrolytes is described.

**Fig. 12:**
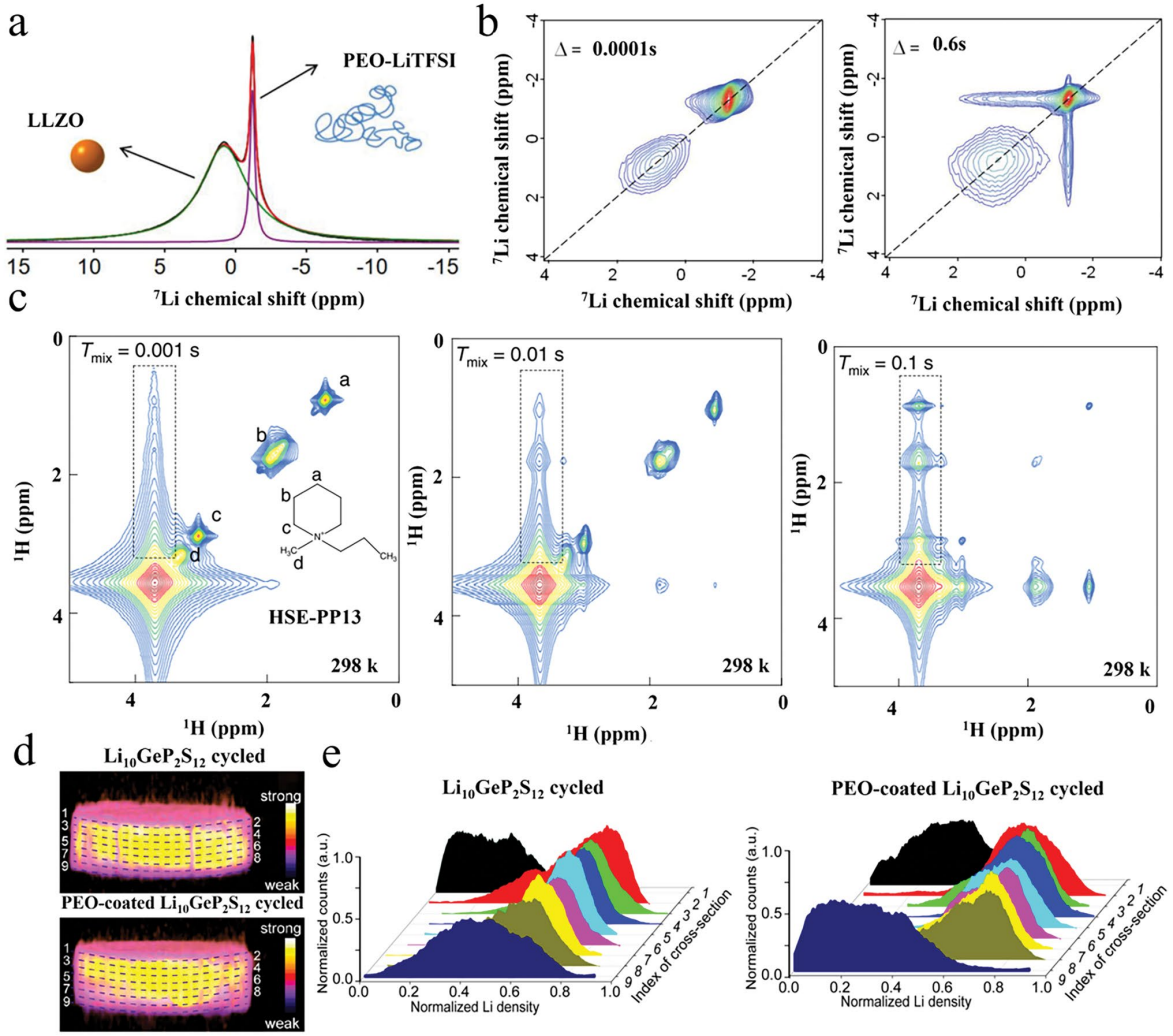
**a** ^7^Li MAS NMR spectra of PEO(LiTFSI)-LLZO OICSE. **b** ^7^Li 2D EXSY NMR spectrum with mixing times of 0.0001 s and 0.6 s, respectively [248], Copyright 2019, American Chemical Society. **c** 2D ^1^H–^1^H NOESY spectra of the mixtures of LiTFSI-PEO-Li_6_PS_5_Cl with PP13-TFSI ILs measured with t_*mix*_ of 0.001, 0.01 and 0.1 s. [249], Copyright 2022 Marnix Wagemake. **d** ^7^Li 3D MRI images of the electrochemically cycled Li_10_GeP_2_S_12_ and PEO-coated Li_10_GeP_2_S_12_ electrolyte. **e** Histograms of normalized Li density at different depths of the cycled Li_10_GeP_2_S_12_ and PEO-coated Li_10_GeP_2_S_12_ electrolyte, respectively [250], Copyright 2018, American Chemical Society

Solid-state NMR spectroscopy is a formidable investigative technique for probing the local structural environments and the dynamics process of lithium ions within ASSLBs. Its high-resolution capabilities allow it to distinguish between lithium ions in different structural environments in the OICSE, including inorganic, polymer, and interface. The ^6^Li-^7^Li isotope tracer technique enables the revelation of Li-ion transport pathways, details of which have been presented in the chapter on ion conduction mechanisms. Additionally, 2D exchange spectroscopy (EXSY) is employed to investigate ion exchange interactions between different phases. The ^7^Li NMR spectra (Fig. 12a) demonstrate the different Li-ion environments. Specifically, the resonance at − 1.2 ppm indicates Li in the polymer matrix, whereas the resonance at 0.8 ppm is representative of the Li in the LLZO [248]. The ^19^F NMR spectra show only a signal, suggesting a lack of interaction between the TFSI^−^ -anion and the LLZO surface. In addition, the ^7^Li 2D NMR EXSY spectra obtained at different mixing times and displayed in Fig. 12b, especially at a mixing time of 0.6 s, where the appearance of the cross-peaks further confirms the chemical exchange of lithium ions across the PEO (LiTFSI) and LLZO phases. Wagemaker et al. employed 2D ^1^H-^1^H nuclear Overhauser effect spectroscopy (NOESY) to understand the function of ionic liquids (ILs) in facilitating the activation of the LiTFSI-PEO-Li_6_PS_5_Cl interface (Fig. 12c) [249]. The observation of cross-peaks between EMIM-TFSI and LiTFSI-PEO at comparable mixing times indicates a lack of specific orientation for EMIM-TFSI relative to PEO, which corroborates their compatibility and the dynamics of EMIM-TFSI within the composite matrix. For HSE-PP13, as shown in Fig. 12c, the prompt manifestation of ^1^H–^1^H correlations between the ^1^H resonances at the 'a' and 'b' positions on the piperidine ring of PP13-TFSI and the –OCH_2_– protons of PEO at abbreviated mixing times suggests a more interactive interface between these components. Notably, these ring protons are located farthest from the bulky propyl and methyl groups attached to the N atom on the piperidine ring. This observation suggests that the positively charged N atom on the piperidine ring and its associated functional groups are positioned away from the PEO segments. The authors subsequently probed the interfacial environment of the two OICSEs employing 2D ^1^H–^6^Li HETCOR spectroscopy. For HSE-EMIM, pronounced correlation signals between PEO and LiTFSI were detected, reflecting the effective solvation of EMIM within the PEO matrix. Conversely, the HSE-PP13 electrolyte exhibited no correlation between PEO and LiTFSI or decomposed Li_6_PS_5_Cl components, indicating a lack of homogeneous miscibility between PP13 and PEO. Further analysis of the PEO-Li_6_PS_5_Cl interface was conducted using ^1^H–^7^Li cross-polarization (CP) experiments, which revealed the proximity of protons to both HSE-PP13 and HSE-EMIM near the Li_6_PS_5_Cl interface.

MRI, a non-invasive imaging technique, has recently been adapted for solid-state lithium metal battery applications. Hu et al. utilized 3D ^7^Li MRI images to capture the edge views of Li_10_GeP_2_S_12_ and PEO-coated Li_10_GeP_2_S_12_ electrolytes (Fig. 12d) [250]. This approach was employed to investigate the lithium distribution within symmetric battery cells after cycling, providing insights into the spatial dynamics of lithium ions during battery operation. The results indicate a localized lithium depletion at the interfacial region, exacerbating the non-uniformity of lithium-ion distribution. Quantitative 3D ^7^Li MRI imaging revealed substantial lithium-ion depletion within the top and bottom layers of the cycled images, demonstrating significant Li-ion losses in the top and bottom layers of cycled Li_10_GeP_2_S_12_ particles, with a more acute deficit observed in the top layer. In comparison, the PEO-coated Li_10_GeP_2_S_12_ electrolyte exhibited a diminished and more evenly distributed lithium loss across both the top and bottom interfaces. Upon examination of the histogram scatter plots detailing lithium content (Fig. 12e), the researchers discerned a non-uniform distribution of lithium in the top and bottom layers of the uncoated samples. Notably, applying a PEO coating mitigated this loss and uneven distribution of lithium ions.

MRI provides intricate insights into the internal structure and interfacial characteristics of OICSEs but still faces many challenges. Primarily, the OICSEs consist of multiple components, including polymers, fillers, interface, solvents, etc., and the MRI signals of these different components may overlap, leading to complex and difficult interpretation of imaging results. Furthermore, investigating microscopic structural details and interfacial attributes within OICSEs requires high-resolution MRI, where the signal is widened through various interactions. However, the existing MRI techniques may not satisfy the requirements for the desired high resolution. Additionally, the high electric and strong magnetic fields generated under battery operating conditions may interfere with MRI imaging, affecting imaging quality and accuracy. Although MRI has a wide range of application prospects in the field of OICSEs, a series of technical difficulties, including signal separation, resolution enhancement, and interference elimination, must be overcome to achieve more accurate and detailed OICSE imaging. To improve the resolution of in-situ NMR techniques for solid-state lithium metal batteries, several aspects should be considered: (1) To achieve simultaneous NMR acquisition while the battery is electrochemically cycling, in-situ NMR requires home-made cells that are adapted to the NMR coil and signal accumulation; (2) To improve the NMR techniques for high efficiency and high spectral resolution; (3) To develop a stronger pulsed field gradient to achieve better spatial resolution in imaging and diffusion determination within the composite electrolyte or polymer electrolyte.

### Time-of-Flight Secondary Ion Mass Spectrometry

TOF–SIMS is a highly sensitive technique for analyzing surface characteristics and elemental compositions. It generates secondary ions by bombarding the sample surface with ions and then obtains information about the chemical composition, molecular structure, and distribution of elements by measuring the flight time of these secondary ions. TOF–SIMS is widely used for the detailed examination and characterization of the chemical composition of the electrolyte/electrode interface due to its desirable properties such as high spatiotemporal and mass resolution. It is particularly suitable for studying the dynamic evolution of electrolyte/electrode surface species such as reaction products and reaction intermediates. This technique can provide a multi-dimensional characterization of the electrolyte/electrode interface and thus reveal electrochemical reaction mechanisms.

**Fig. 13:**
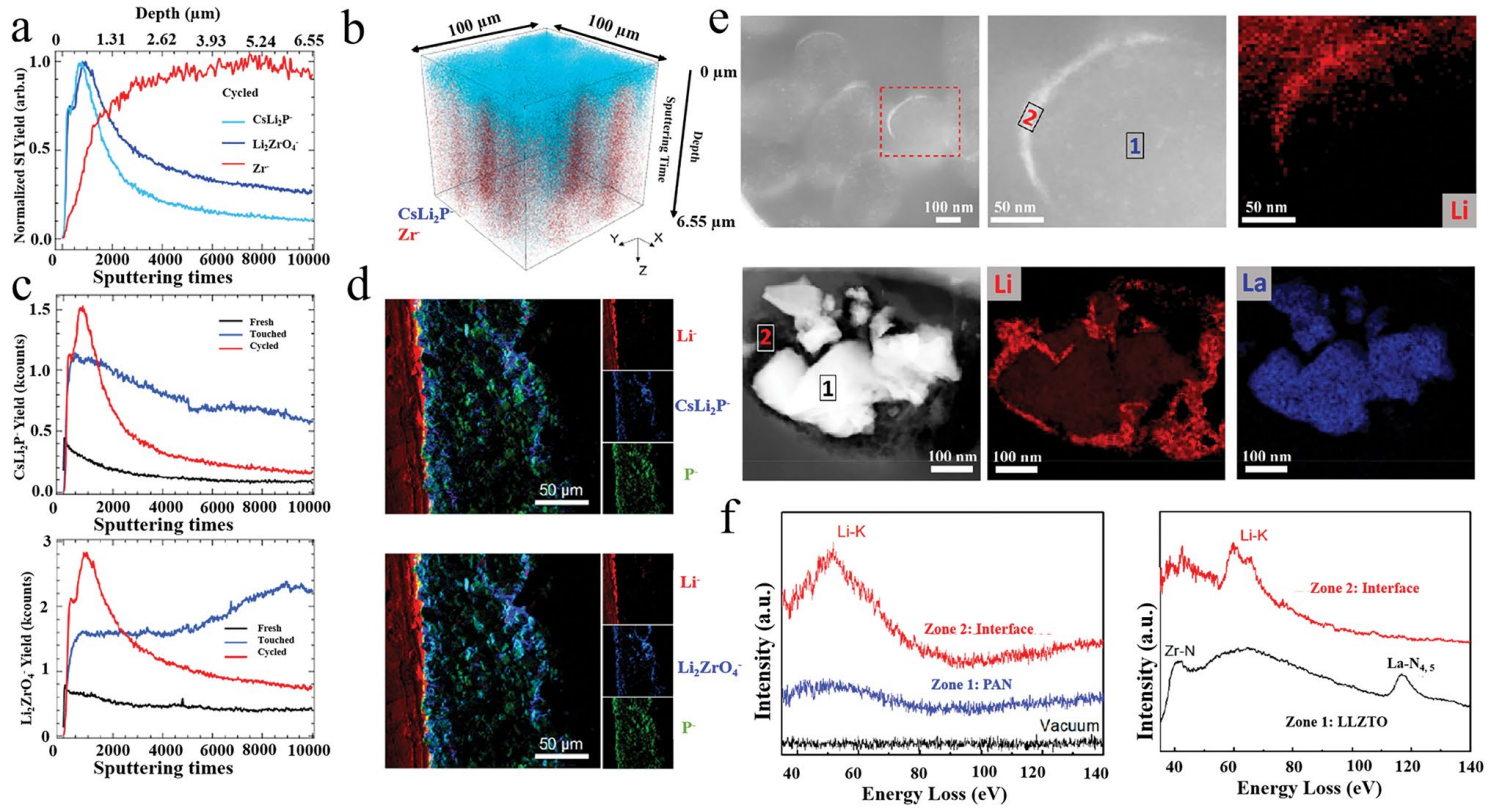
**a** Normalized TOF–SIMS depth profiles of CsLi_2_P^−^, Li_2_ZrO_4_^−^, and Zr^−^, representing Li_3_P and Li_8_ZrO_6_ reacted species, and bulk LiZr_2_(PO_4_)_3_, respectively. **b** 3D view of the sputtered volume in panel a. **c** A direct comparison of CsLi_2_P^−^ and Li_2_ZrO_4_^−^, depth profiles obtained from the fresh composite membrane, the composite membrane after interaction with lithium metal, and the composite membrane after cycling the Li/Li symmetric cell. **d** TOF–SIMS high-resolution secondary ion maps of a Li/electrolyte cross-section [85], Copyright 2020, American Chemical Society. **e** HAADF-TEM images of PAN/LiClO_4_ and the PAN/LiClO_4_: LLZTO and corresponding EELS element concentration distribution map. **f** EELS spectra of selected regions of organic particle phase, organic/organic interface and polymer phase, polymer/inorganic interface [215], Copyright 2020, American Chemical Society

Goodenough et al. employed depth profiling and cross-sectional imaging via TOF–SIMS to investigate the interface between Li anode and CPE-25LZP electrolytes [85]. Figure 13a illustrates the surface concentration of CsLi_2_P^−^ and Li_2_ZrO_4_^−^ species, using Zr^−^ as an indicator for the bulk solid electrolyte after cycling in a Li/Li symmetric cell. The 3D representation of the sputtered volume, shown in Fig. 13b, illustrates the spatial distribution of CsLi_2_P^−^ and Zr^−^ signals. Significantly, beyond the surface concentration of CsLi_2_P^−^, a fragmented distribution of both CsLi_2_P^−^ and Zr^−^ ions is evident, indicating the particulate nature of the solid electrolyte. Figure 13c displays a comparative analysis of depth profiles for CsLi_2_P^−^ and Li_2_ZrO_4_^−^ in three states: the fresh composite membrane, the composite membrane post-lithium metal interaction, and the composite membrane after cycling in Li||Li symmetric cell. The data reveal a significant increase in CsLi_2_P^−^ and Li_2_ZrO_4_^−^ concentrations at the surface of membranes that have either been cycled or exposed to lithium compared to the fresh membrane. For a direct examination of the chemical composition along the Li/solid electrolyte interface, the authors conducted cross-sectional mapping with high lateral resolution, as depicted in Fig. 13d. This mapping identified the presence of CsLi_2_P^−^ and Li_2_ZrO_4_^−^ species at the interface, substantiating the particulate character of the solid electrolyte at the micrometer level.

TOF–SIMS provides unprecedented spatial, temporal, and mass resolution for monitoring electrochemical processes. However, achieving the goal of visualizing complex and dynamic multiphase electrochemical processes remains a major challenge. TOF–SIMS has unprecedented high mass resolution and high spatial resolution in monitoring electrochemical processes, but achieving the goal of visualizing complex and dynamic multiphase electrochemical processes remains challenges. TOF–SIMS requires a high vacuum environment to ensure the long free range of secondary ions, which poses significant challenges for complex multiphase interfaces (including liquid and solid interfaces). Therefore, there is a need to develop advanced measurement methods and vacuum-compatible electrochemical microfluidic devices to better understand dynamic electrochemical processes. In addition, the high energy of the primary ion beam can damage interfacial species, affecting the accuracy of analytical results. New primary ion sources need to be developed to improve the yield and mass resolution of secondary ions with both spatial and temporal resolution.

### High-Angle Annular Dark-Field Scanning Transmission Electron Micrographs and Electron Energy Loss Spectroscopy

Chen et al. utilized HAADF-STEM and EELS techniques to confirm that lithium preferentially accumulates at polymer/polymer and polymer/inorganic interfaces [215]. As shown in Fig. 13e, the Li element accumulation was observed at the periphery of PAN fiber through STEM-EELS Li K-edge mapping, further verified by EELS spectroscopy at both the PAN fiber interfaces and inner regions. The EELS spectra reveal that lithium-enriched areas surround LLZTO nanoparticles. In the EELS spectra obtained from the LLZTO region (region 1), the broad edge is observed in the 50–80 eV (Fig. 13f), lacking distinctive features. By contrast, the La-N_4,5_ edge appears at approximately 110 eV, indicating a disordered chemical environment for the Li ions in LLZTO. Conversely, the spectra from the interfacial region (region 2) exhibit a more defined double-peak characteristic of the Li K-edge, suggesting a more homogeneous coordination environment. Consequently, high-resolution transmission electron microscopy (HR-TEM) imaging via HAADF-STEM facilitates the visualization of the microstructure and component distribution within the material. This approach enables the observation of the distribution, morphology, and interfaces of various components in the material, thereby enhancing our understanding of the internal structure of the composite electrolyte. Combining with EELS to analyze the elemental composition and electronic structure of material contributes to optimizing the design and performance enhancement of the composite electrolyte.

HAADF-STEM can provide high-resolution images at the sub-nanometer scale to observe the nanostructures and interfaces in composite electrolytes. Through electron tomography, HAADF-STEM realizes three-dimensional reconstruction of composite electrolyte materials, which helps to study their internal structure and morphology. Combined with EELS, HAADF-STEM can perform elemental distribution and chemical state analysis, providing detailed information about the electronic structure and chemical bonding composition in composite electrolyte materials. However, high-energy electron beams can cause damage to some sensitive materials (e.g., organic or some inorganic complexes), resulting in structural changes or decomposition that may affect the observation results. Since the imaging area is very small, the results may not be representative of the homogeneity and macroscopic properties of the entire composite electrolyte material. Therefore, a more comprehensive understanding of the properties of composite electrolyte materials can be obtained by combining it with other characterization methods.

### Small-Angle X-Ray Scattering (SAXS)

**Fig. 14:**
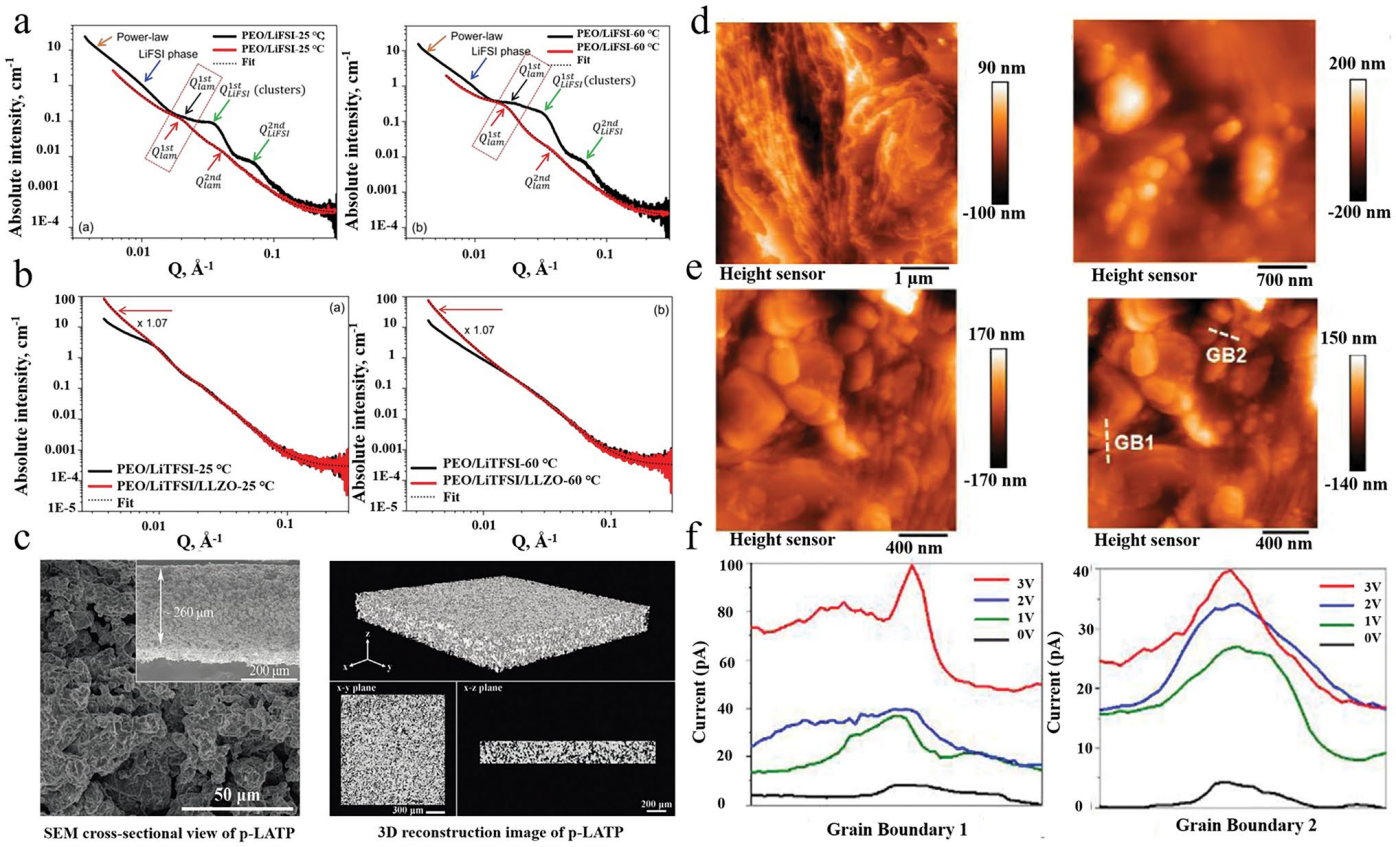
**a** SAXS curves of the pristine PEO (without Li salts) and PEO/LiFSI electrolytes at 25 and 60 °C. **b** SAXS curves of PEO/LiTFSI/LLZO were multiplied by 1.07 (~ 1/0.93) to normalize the scattering intensity for the less fraction of PEO/LiTFSI due to the added 0.07 (7%) LLZO [251] Copyright 2024, American Chemical Society. **c** SEM cross-sectional view of p-LATP, 3D reconstruction image of p-LATP and corresponding 2D sliced images from x–y, x–z plane [252] Copyright 2021, Wiley‐VCH GmbH. **d** In-situ* c* -AFM characterization of Li-ion migration in pure PEO(LiClO_4_) and 50 wt% LLZO-PEO(LiClO_4_) at 55 °C.** e** In-situ c-AFM characterization of Li-ions migration in 75 wt% LLZO-PEO(LiClO_4_) at 30 and 55 °C. **f** c-AFM current curve at grain boundaries, where grain boundary 1 and grain boundary 2 [253] Copyright 2021 Elsevier B.V

Li et al. have thoroughly investigated the interaction between different lithium salts (LiFSI and LiTFSI), LLZO, and PEO and the effect on the nanostructure and ion transport behavior by SAXS [251]. The SAXS curves of pure PEO, PEO/LiFSI, PEO/LiTFSI, PEO/LiTFSI/LLZO at 25 and 60 °C are shown in Fig. 14a, b. At 25 and 60 °C, pure PEO exhibits first-order and second-order scattering peaks (red arrows) characteristic of the periodic layered structure formed by PEO in spherical crystals. In PEO/LiFSI, the SAXS curves show scattering peaks like pure PEO, but there are additional scattering features (black arrows) associated with the spherical clusters of LiFSI (Fig. 14a). The average radii of LiFSI clusters in the PEO/LiFSI electrolyte were about 8.5 and 8.7 nm at 25 and 60 °C, while the localized volume fractions were 0.35 and 0.28, respectively, and the localized volume fractions at 60 °C indicated that some LiFSI clusters dissolved and diffused into the amorphous PEO phase. In PEO/LiTFSI, on the other hand, the first and second peaks completely disappeared at 60 °C, indicating that LiTFSI clusters were completely dissolved in the PEO matrix. Therefore, it can be assumed that LiTFSI is more easily dissolved in PEO than LiFSI. Comparing the SAXS curves of PEO/LiTFSI and PEO/LiTFSI/LLZO (Fig. 14b), it is found that the scattering intensity of PEO/LiTFSI/LLZO is significantly increased in the low Q region, which is attributed to the addition of the micrometer-sized LLZO, whose size is beyond the measurable range of SAXS. In the higher Q region, the SAXS curves almost overlapped, indicating that the addition of LLZO did not change the microstructure of PEO and LiTFSI.

The analysis demonstrates the powerful application of SAXS in studying microstructures and interactions in polymer electrolyte or composite electrolyte systems. However, SAXS is suitable for studying structures in the range from 1 to 100 nm, and its resolution is significantly reduced for very small atomic-scale structures or larger micrometer-scale structures. SAXS relies on the difference in electron density between different components. It is difficult for SAXS to distinguish the microstructure of components with similar electron densities, such as some organics and polymers. In addition, when studying composite electrolytes, the presence of different phases increases the complexity of data interpretation, and the scattering characteristics of each phase may overlap with each other, making it difficult to resolve them individually. Choosing the right model and fitting it correctly is a challenge, especially in multi-component systems.

### X-Ray Computed Tomography (CT)

CT technology provides a non-destructive, 3D imaging method to gain insight into the internal microstructure of OICSEs. This is important for understanding the relationship between the microstructure and macroscopic performance of composite electrolytes, optimizing material design, and improving the performance of solid-state batteries [254]. The CT technology can determine the three-dimensional distribution of organic and inorganic phases in a composite electrolyte, measure the porosity of the composite electrolyte, and analyze the morphology, size, and distribution of the pores. Cui et al. [252] presented the self-supported highly porous p-LATP with a thickness of about 260 μm using SEM imaging and further revealed the porous microstructure of p-LATP by CT (Fig. 14c). The uniform pore distribution observed in the 3D reconstructed images of p-LATP and the corresponding 2D sliced images in the x–y and x–z planes demonstrate p-LATP with percolated porous structure has been fabricated successfully. In addition, the 3D images allow the study of pore connectivity and its effect on ionic conduction, as well as the observation of the evolution of defects in the composite electrolyte during use, helping to understand the lifetime and stability of the material. The development of high-power solid-state batteries can be critically supported by CT technology. However, compared to nano-CT, the resolution of CT is low, usually in the micrometer range (about 1–100 µm), which is suitable for larger-scale imaging.

### Atomic Force Microscopy (AFM)

Liu et al. prepared LLZO-PEO composite electrolytes with different weight ratios (0, 50, and 75 wt%) and used in situ conduction atomic force microscopy (c-AFM) to observe the changes in the morphology and mechanical properties of the electrolytes at different temperatures [253]. At 30 °C, most of the PEO in pure PEO (LiClO_4_) was in a crystalline state, and when the temperature was increased to 55 °C, most of the PEO changed from crystalline to amorphous, with only a small amount of chain-like crystalline PEO. At 55 °C for the 50 wt% LLZO-PEO sample, the transformation of the chained PEO into an amorphous state can be observed (Fig. 13d). At 30 °C, many LLZO particles can be observed in the AFM topography, and the currents are still concentrated in the amorphous PEO region, with no currents observed inside the LLZO crystal. However, when the temperature is increased to 55 °C (Fig. 14e), currents of the same order of magnitude are observed not only between the LLZO phases but also inside the LLZO crystal. LLZO and PEO can be easily distinguished based on Young's modulus and adhesion. Figure 14f shows the current response in the voltage range of 0–3 V at the grain boundaries, where the current values vary considerably due to the position of the LLZO particles in the electrolyte. This indicates that despite the presence of a large amount of LLZO, PEO remains crystalline at low temperatures and lithium ions cannot migrate rapidly through the LLZO network. However, when a large amount of LLZO is added, it breaks the crystalline chain of PEO and forms a continuous ion migration network in the PEO matrix, and lithium ions can migrate along the LLZO network at high temperatures. However, excess LLZO can accumulate in the matrix and reduce the transport efficiency of lithium ions.

AFM can provide sub-nanometer spatial resolution and, in addition to imaging, can measure mechanical properties of materials (e.g., hardness, Young's modulus). Sample preparation requirements are low, no special treatment of the sample is required, and in-situ observation can be performed directly in different environments (e.g., vacuum, liquid, gas) and under different conditions (e.g., temperature, humidity changes) to monitor changes in the sample in real-time. However, AFM scanning speed is relatively slow, especially in high-resolution mode, and it can take several hours to image a large sample area. Observations can only be made on the surface, with limited ability to examine samples with large thicknesses or internal structures. In addition, the probe wears out after a long time, and the interaction force between the probe and the sample must be precisely controlled to avoid damaging the sample or the probe, as well as to ensure the quality of the image and the accuracy of the measurement.

Several commonly utilized techniques are employed to characterize the physicochemical properties of OICSEs. These include X-ray diffraction (XRD) and neutron scattering (NDP) for analyzing crystal structure and phase properties and X-ray photoelectron spectroscopy (XPS) for investigating surface chemical states and elemental distribution, thermal analysis techniques (DSC and TGA) are employed to explore the thermal properties and stability of the electrolyte. Electrochemical impedance spectroscopy (EIS) measures the conductivity and interface properties of the electrolyte, while Raman spectroscopy and infrared spectroscopy are utilized to analyze the molecular vibrational modes of the materials. Integrating these diverse characterization techniques facilitates a comprehensive and detailed understanding of the performance and structure of OICSEs.

## Summary and Perspective

ASSLBs are widely acknowledged as the most promising next-generation energy storage systems owing to their intrinsic safety features and remarkable energy density. This review highlights the development of OICSEs, which are pivotal in SE advancements due to their integration of various electrolytic components. We critically analyze the essential parameters for assessing OICSE performance, including ionic conductivity, Li^+^ transference number, mechanical properties, electrochemical stability, electronic conductivity, and thermal stability. We explore the impact of ceramic fillers on ionic conductivity, considering factors like particle size, content, shape, and dimension. The review also investigates Li^+^ transport mechanisms and the role of different inorganic fillers, both inert (0D particles, 1D nanowires, 2D nanosheets, 3D frameworks) and active (sulfide, garnet, NASICON, and perovskite types) in enhancing OICSE performance. Finally, the importance of advanced characterization techniques for OICSEs is emphasized, as they provide a more comprehensive understanding of the chemical composition, microstructure, and interfacial properties. Despite significant advancements in the research of OICSEs, the technological maturity of ASSLBs utilizing these electrolytes remains insufficient for practical application and commercialization. Therefore, considering the challenges presented by OICSEs in ASSLBs, this review identifies and proposes potential avenues for future research and development.

Despite the improvement in ionic conductivity in previous OICSEs, some significant challenges remain. Presently, the ionic conductivity of most OICSEs lingers in the range of 10^−4^ to 10^−5^ S cm^−1^, remaining inferior to that of liquid electrolytes (~ 10^−2^ S cm^−1^). Future strategies to augment ionic conductivity could be directed toward several critical areas of development. (1) Combining different scales of fillers and electrolytes, designing multi-stage pore structure (e.g., 3D LLTO/LATP/LLZO), optimizing ion transport channels from micro to macro level, thereby enhancing overall conductivity. (2) Controlling and adjusting thin film thickness can reduce ion transport distances (Commonly used technologies, including electrostatic spinning, osmotic hot pressing, and combination electrospinning/electrospray technology). Meanwhile, ensuring sufficient mechanical strength and flexibility is crucial to prevent lithium dendrite growth. (3) Introducing of materials rich in surface functional groups (e.g., MXene-Ti_3_C_2_ and BN with functional groups such as –OH, –O, –NH_2_, and –F.), as well as surface modification (e.g., PDA or DMSO modification of LLZTO nanoparticles), etc., to improve the homogeneity of the filler dispersion. (4) Exploring new materials with high Li^+^ transference numbers, including polymers with elevated ion mobility, conductive polymers, and inorganic electrolytes. (5) Employing intelligent design and simulation, leveraging computational simulation, machine learning, and other advanced technologies to design OICSEs.

Due to multiple complex Li^+^ transport mechanisms, the precise mode of Li-ion transport within these systems remains unclear. Nonetheless, understanding the interactions is crucial for advancing ionic conductivity. Advanced characterization techniques such as solid-state NMR and in-situ characterization methods can be employed to observe variations in ion migration capacity and concentration within OICSEs under different conditions. Moreover, integrating computational approaches, including molecular dynamics simulations and density functional theory, facilitates a thorough investigation of ion transport mechanisms at multiple scales. These simulations are instrumental in predicting ion diffusion pathways and elucidating the impact of interface interactions. Furthermore, tailoring interfaces, namely designing specific interfaces between polymer components and fillers, enables precise control over ion transport channels. Moreover, researchers can harness synergistic effects to enhance ionic conductivity by leveraging the various ion transport mechanisms in different components of OICSEs.

The interface in OICSEs primarily encompasses two aspects: between the polymer and fillers and between the OICSEs and electrodes. Optimizing interface stability is crucial for improving the performance and extending the cycle life of ASSLBs. The disparate chemical properties and interactions between polymers and inorganic fillers often lead to interfacial compatibility issues. To address this issue, surface modification techniques that introduce appropriate functional groups have been implemented to enhance compatibility with the polymer matrix, consequently promoting the uniform dispersion of nanoparticles. Additionally, incorporating small molecule plasticizers, such as SN, has proven effective in enhancing interfacial compatibility. The contact between OICSEs and electrodes can lead to electrochemical reactions, triggering interfacial degradation and instability. In situ polymerization can create a uniform, continuous, and dense interfacial layer on the electrode surface, effectively reducing interfacial resistance, which is one of the critical development directions. In the field of ASSLBs, a pivotal objective is the development of OICSEs that exhibit resistance to high voltages, improve compatibility with high-voltage cathodes, and effectively protect the interface from electrochemical reactions and degradation. Furthermore, employing special coating technologies (e.g., OICSEs reacting with Li metal to produce in situ protective layers such as LiF, or introducing polymer buffer layers such as PVDF and PEO) can improve the intimate contact between OICSE and electrodes or enhance interaction through the innovative design of electrode structures. Through the optimization of the OICSEs interface, a more efficient and stable energy storage device can be achieved, thereby promoting the development and practical application of battery technology.

More detailed and accurate information on OICSEs can be obtained through advanced characterization techniques. This offers valuable insights into the internal interfacial structure, composition, properties, and behavior of these materials. Nonetheless, these techniques face significant challenges, including balancing high resolution with rapid data acquisition. Increasing data acquisition speed may reduce resolution while enhancing resolution can slow down the process. Additionally, these techniques often generate large volumes of data, complicating the extraction of valuable insights and precise analysis. Integrating experimental results with theoretical models is essential for accurately characterizing samples. Another challenge is that numerous advanced techniques demand specialized sample handling or preparation, encompassing procedures such as cutting, grinding, coating, etc. Therefore, future directions include integrating different characterization techniques to provide more comprehensive information. Further development of techniques for in-situ observation and atomic-level resolution will contribute to a deeper understanding of material behavior and changes under different conditions. Simultaneously, refining non-invasive characterization techniques, especially for precious samples, becomes important to prevent sample damage.

Roll-to-roll processing for solid-state batteries is a continuous, high-productivity manufacturing technology suitable for large-scale production, which is similar to the large-scale continuous roll-to-roll process used to manufacture conventional lithium-ion batteries, but the process needs to be adapted to handle solid-state materials and ensure compatibility with high-voltage cathodes and lithium metal anodes. Roll-to-roll processing significantly reduces manufacturing costs by reducing material waste and improving production efficiency and is suitable for manufacturing large-area and flexible electronic devices. The Central Research Institute of Electric Power of Japan has prepared a two-layer polymer battery cell using roll-to-roll technology with an output voltage of 12 V. The positive electrode is LiNi_1/3_Mn_1/3_Co_1/3_O_2_ with a potential of more than 4 V, the negative electrode is graphite, and the solid electrolyte is a polyether material. The 3D printing technology, with a high degree of design freedom and rapid prototyping, is suitable for producing personalized and complex structures, realizing novel structures that are unattainable by traditional methods, thus enhancing ionic conductivity and mechanical stability. Despite the advantages of 3D printing technology in customizing battery components, printing accuracy still needs to be improved and is currently relatively expensive. However, the cost issue is expected to be alleviated as the technology continues to advance. Thus, both roll-to-roll machining and 3D printing technologies offer promising paths to scalable and cost-effective manufacturing of solid-state batteries.
